# Could Immune Checkpoint Disorders and EBV Reactivation Be Connected in the Development of Hematological Malignancies in Immunodeficient Patients?

**DOI:** 10.3390/cancers15194786

**Published:** 2023-09-29

**Authors:** Paulina Mertowska, Sebastian Mertowski, Konrad Smolak, Gabriela Kita, Katarzyna Guz, Aleksandra Kita, Marcin Pasiarski, Jolanta Smok-Kalwat, Stanisław Góźdź, Ewelina Grywalska

**Affiliations:** 1 Department of Experimental Immunology, Medical University of Lublin, 20-093 Lublin, Poland; paulinamertowska@umlub.pl (P.M.); smolakkonrad93@gmail.com (K.S.); gabikita1@gmail.com (G.K.); kasia08263@gmail.com (K.G.); olakita00@gmail.com (A.K.); ewelina.grywalska@umlub.pl (E.G.); 2 Student Research Group of Experimental Immunology, Medical University of Lublin, 20-093 Lublin, Poland; 3 Department of Immunology, Faculty of Health Sciences, Jan Kochanowski University, 25-317 Kielce, Poland; marcinpasiarski@gmail.com; 4 Department of Hematology, Holy Cross Cancer Centre, 25-734 Kielce, Poland; jolantasmok1@gmail.com (J.S.-K.); stanislawgozdz1@gmail.com (S.G.); 5 Institute of Medical Science, Collegium Medicum, Jan Kochanowski University of Kielce, IX Wieków Kielc 19A, 25-317 Kielce, Poland

**Keywords:** immunodeficiency, Epstein–Barr virus (EBV), PD-1, PD-L1, CTLA-4, CD86, CD200R, CD200, immune checkpoints, common variable immunodeficiency (CVID), chronic lymphocytic leukemia (CLL), cancer risk

## Abstract

**Simple Summary:**

Primary immunodeficiencies (PIDs) and secondary immunodeficiencies (SIDs) weaken the immune system, making people prone to infections and possibly affecting cancer development. Epstein–Barr virus (EBV), a common virus, is linked to cancer, especially in those with weak immunity. This study compares immune factors like PD-1/PD-L1, CTLA-4/CD86, CD200R/CD200, and EBV in chronic lymphocytic leukemia (CLL, a SID) and common variable immunodeficiency (CVID, a PID). We studied CLL, CVID, and healthy people, checking EBV activity and immune checkpoints. Both CLL and CVID patients showed more EBV activity, and their immune checkpoints were changed, possibly affecting EBV and immunity. This study shows how immune issues, EBV, and checkpoints might contribute to cancer in people with weakened immunity. It suggests ways to manage these risks. More research is needed to understand this fully and develop treatments.

**Abstract:**

Primary immunodeficiencies (PIDs) and secondary immunodeficiencies (SIDs) are characterized by compromised immune function, rendering individuals susceptible to infections and potentially influencing cancer development. Epstein–Barr virus (EBV), a widespread herpesvirus, has been linked to cancer, particularly in those with weakened immune systems. This study aims to compare selected immune parameters, focusing on immune checkpoint molecules (PD-1/PD-L1, CTLA-4/CD86, CD200R/CD200), and EBV reactivation in patients with chronic lymphocytic leukemia (CLL, a representative of SIDs) and common variable immunodeficiency (CVID, a representative of PIDs). We performed a correlation analysis involving patients diagnosed with CLL, CVID, and a healthy control group. EBV reactivation was assessed using specific antibody serology and viral load quantification. Peripheral blood morphology, biochemistry, and immunophenotyping were performed, with emphasis on T and B lymphocytes expressing immune checkpoints and their serum concentrations. Our findings revealed elevated EBV reactivation markers in both CLL and CVID patients compared with healthy controls, indicating increased viral activity in immunodeficient individuals. Furthermore, immune checkpoint expression analysis demonstrated significantly altered percentages of T and B lymphocytes expressing PD-1/PD-L1, CTLA-4/CD86, and CD200R/CD200 in CLL and CVID patients. This suggests a potential interplay between immune checkpoint dysregulation and EBV reactivation in the context of immunodeficiency. In conclusion, our study underscores the intricate relationship between immune dysfunction, EBV reactivation, and immune checkpoint modulation in the context of immunodeficiency-associated cancers. The altered expression of immune checkpoints, along with heightened EBV reactivation, suggests a potential mechanism for immune evasion and tumor progression. These findings provide insights into the complex interactions that contribute to cancer development in immunocompromised individuals, shedding light on potential therapeutic targets for improved management and treatment outcomes. Further investigations are warranted to elucidate the underlying mechanisms and to explore potential interventions to mitigate cancer risk in these patient populations.

## 1. Introduction

Primary immunodeficiencies (PIDs) and secondary immunodeficiencies (SIDs) are conditions that affect the immune system’s ability to protect the body from infections and can potentially affect the development of cancer [1,2,3,4]. PIDs are inherited genetic disorders that cause defects in various components of the immune system. These defects can lead to increased susceptibility to infections, autoimmune diseases, and, in some cases, an increased risk of cancer [5,6,7,8]. Some PIDs are associated with a higher risk of certain cancers, mainly due to impaired immune surveillance that normally helps detect and eliminate cancer cells [9,10]. One such example is common variable immunodeficiency (CVID). CVID is an immune disorder characterized by low levels of serum immunoglobulins and antibodies, leading to increased susceptibility to infection and a higher risk of autoimmune diseases, cancers, and lymphoproliferative disorders (like lymphomas, gastric cancer, and other malignancies) [11,12,13,14]. The pathogenesis of CVID is not fully understood, but it is associated with various abnormalities in B cell differentiation and function. Recent studies suggest that dysregulation of the immune checkpoint may also be involved [15,16,17,18,19].

SIDs are acquired disorders that result from external factors, such as infections, medications, and other medical conditions, and can also affect the immune system’s ability to function properly [3,20,21]. These factors can sometimes contribute to an increased risk of cancer. One example of a SID that is also an example of a hematologic malignancy is chronic lymphocytic leukemia (CLL). CLL is a type of cancer where the bone marrow produces too many lymphocytes (a type of white blood cell), often leading to dysfunction of the immune system, which the literature suggests may be related to checkpoint molecules such as PD-1 (programmed cell death 1) and its ligand PD-L1, or CTLA-4 (T cell cytotoxic antigen 4), which may play a significant role in the progression of CLL. Overexpression of these inhibitory molecules often leads to immune exhaustion and the formation of a suppressive tumor microenvironment, allowing malignant cells to escape immune surveillance [22,23,24,25,26,27,28,29].

It is important to remember that while immunodeficiency may contribute to an increased risk of certain cancers, not everyone with a PID or SID will develop cancer. The interplay between the immune system and cancer development is complex and depends on various factors, including genetic predisposition, environmental exposure, and the specific nature of the immune defect [30,31]. Scientists are increasingly elevating the role of two rather extreme but mutually interacting mechanisms: the deregulation of immune checkpoints and the role of Epstein–Barr virus (EBV) reactivation [32,33,34]. EBV belongs to the herpesvirus family and is one of the most common viruses in humans. It is usually associated with causing infectious mononucleosis (commonly known as mononucleosis or glandular fever) in teenagers and young adults [35,36,37]. EBV infection is usually mild and self-limiting, but in some cases, it can lead to more serious complications, especially in people with PIDs or SIDs. In addition, research in recent years has linked EBV to the development of several types of cancer, especially in people with a weakened immune system or certain genetic predispositions [38,39]. EBV-associated cancers are more common in regions with higher rates of EBV infection and are often linked to the ability of the virus to cause latent (dormant) infections in certain cells [40,41]. The interplay between EBV, the immune system, and other environmental and genetic factors is complex and not fully understood. According to researchers, EBV can manipulate the immune system by interfering with immune checkpoint function. For example, latent EBV infection may increase PD-L1 expression, enhancing immune evasion [32,42,43].

Therefore, the purpose of this publication was a comparative analysis of selected parameters of the immune system, with particular emphasis on the role of immune checkpoints and their ligands (PD-1/PD-L1, CTLA-4/CD86, and CD200R/CD200) in the course of patients with CLL (representing a group of with SID) and CVID (representing a group with PID) in the context of EBV reactivation. The immunological checkpoints selected for the analysis, such as PD-1/PD-L1, CTLA-4/CD86, and CD200R/CD200, play a significant role in regulating the immune response in many diseases, including cancer, and influencing the tumor microenvironment. They are responsible not only for regulating the immune response but also for maintaining self-tolerance to prevent excessive immune reactions against healthy tissues. However, cancer cells can use these checkpoints to evade immune surveillance, allowing tumors to grow and spread. Selected immune checkpoints are among the most common targets of anticancer therapies. Testing immune checkpoints in patients with PID and SID could be extremely important in monitoring cancer development, which will be critical to tailoring therapeutic approaches, predicting responses, and advancing our understanding of the complex interactions between the immune system and hematological malignancies in this particular group of patients.

For this purpose, analyses were carried out to assess EBV reactivation (serological profile of specific antibodies and the number of EBV copies in the tested genetic material) among patients with immunodeficiency in relation to healthy volunteers. We focused on selected parameters including morphology, biochemistry, and immunophenotype of peripheral blood of patients included in the study, with particular emphasis on the percentage of T and B lymphocytes positively expressing PD-1/PD-L1, CTLA-4/CD86, and CD200R/CD200, and the assessment of their serum concentrations.

The performed analyses showed a significant increase in the percentage of these checkpoints and ligands in both groups of patients compared with those without EBV. In particular, the PD-1, PD-L1, CTLA-4, CD86, CD200R, and CD200 pathways showed marked upregulation in response to EBV reactivation. Notably, the observed changes were more pronounced in CLL patients with EBV reactivation compared with CVID patients with the same disease. Our studies suggest that EBV reactivation plays a key role in modifying the immune landscape in both CLL and CVID patients.

## 2. Materials and Methods

### 2.1. Characteristics of Participants and Study Materials

A total of 142 participants were enrolled in this study: 60 individuals with chronic lymphocytic leukemia (CLL), 52 individuals with common variable immunodeficiency (CVID), and 30 healthy volunteers (HVs) who formed the control group. Inclusion and exclusion criteria were applied to both immunodeficient patients and HVs. Selection of participants was conducted by a clinical immunology specialist and based on specific conditions: • age 18 or older; • life expectancy of at least 12 months; • no recent immunosuppressive treatment in the three months prior to study entry; and • willingness to provide written consent for study participation. Patients were excluded from the study based on the following criteria:

- Ongoing viral, bacterial, and fungal infections;
- Severe allergies;
- History of hematopoietic cell or organ allotransplantation;
- Ongoing treatment for active malignancy or other autoimmune diseases;
- Pregnancy or lactation;
- Use of investigational drugs;
- Presence of tumor metastases in the central nervous system or mental illness.

In addition, all participants were age-matched for CLL (median age: 57; range: 37–75; 24 women and 36 men); CVID (median age: 55; range: 31–77; 27 women and 25 men); HVs (median age: 54; range: 32–71; 18 women and 12 men). The age of the patients shown is the age at which the diagnosis was made. None of the patients included in this study had undergone any therapy prior to analysis. Patients with CLL were classified according to Rai Stage 0 (36 subjects) and I (24 subjects); the patients did not show splenomegaly or hepatomegaly and had not undergone any therapy at the time of this analysis or before it was performed. This study utilized 10 mL of peripheral blood drawn from the basilic vein into EDTA (Strasted) tubes for immunophenotypic analysis and 5 mL of blood collected in a clot tube to obtain serum for quantitative measurement of the tested molecules. The research protocol received ethical approval from the Bioethics Committee at the Medical University of Lublin (KE-0254/186/06/2023).

### 2.2. Quantification of EBV Genomic Copies in PBMC-Derived DNA

EBV genomic copy quantification was conducted utilizing the ISEX variant of the EBV PCR assay by GeneProof (Brno, Czech Republic). Duplicate assessments were carried out for all samples, with the inclusion of a negative control containing DNA elution buffer. Amplification targeted the conserved DNA sequence specific to the EBNA1 gene of EBV, using the 7300 Real-Time PCR System (Applied Biosystems, Foster City, CA, USA) in strict accordance with the ISEX EBV PCR kit protocol. The final viral DNA copy concentration, expressed per μg of extracted DNA, was normalized considering the DNA isolation efficiency. A detection threshold was established at ten EBV DNA copies/μL, and samples falling beneath this level were deemed EBV-negative.

### 2.3. Lymphocyte Immunophenotyping

**Figure 1:**
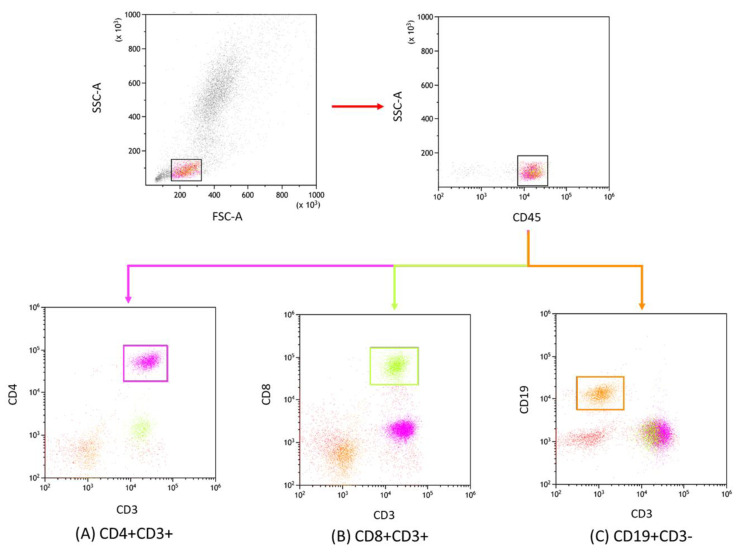
Flow cytometric analysis and gating schema. This illustration depicts the gating strategy implemented in our study to delineate lymphocyte subpopulations. Specifically, the subsets included are (**A**) CD4+ CD3+ (represented in violet), (**B**) CD8+ CD3+ (highlighted in green), and (**C**) CD19+ CD3− (shown in orange). This segregation facilitated the subsequent examination of distinct immunological checkpoint markers within each subset.

**Figure 2:**
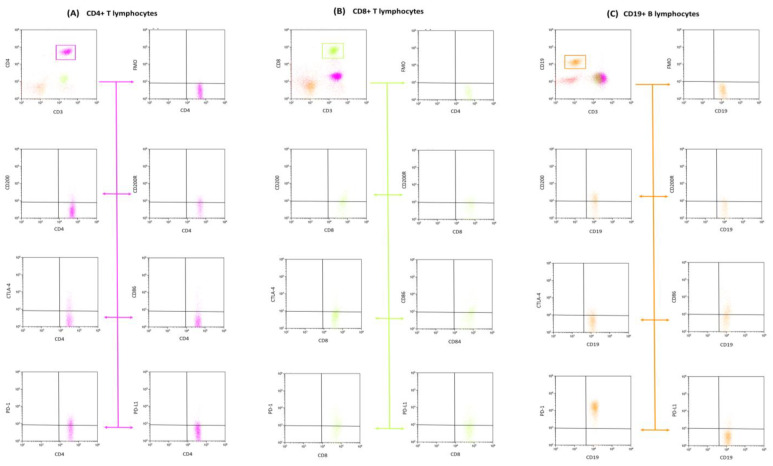
Flow cytometric analysis and gating strategy. This depiction showcases the gating approach utilized to discern subpopulations of T and B lymphocytes. Specifically, we quantified the events expressing CD4+ CD3+ (represented in violet), CD8+ CD3+ (highlighted in green), and CD19+ CD3− (shown in orange) phenotypes. These subpopulations were further examined for expression of molecular markers including CD200, CD200R, CTLA-4, CD86, PD-1, and PD-L1. To bolster the precision of our gating and data interpretation, fluorescence minus one (FMO) controls were incorporated as negative benchmarks, helping to define the optimal gating parameters.

Lymphocyte immunophenotypic profiling from peripheral blood was undertaken utilizing flow cytometry. Blood samples were subjected to a comprehensive panel of human-specific monoclonal antibodies, which encompassed: anti-CD45 AF700 (cat. no.: 368514, clone: 2D1), anti-CD4 BV421 (cat. no.: 300532, clone: RPA-T4), anti-CD3 PerCp (cat. no.: 344814, clone: SK7), anti-CD8 BV605 (cat. no.: 344742, clone: SK1), and anti-CD19 FITC (cat. no.: 302206, clone: HIB19), alongside antibodies targeting immune checkpoints and other relevant markers such as anti-PD-1 APC (cat. no.: 329908), anti-PD-L1 PE (cat. no.: 329706, clone: 29E.2A3), anti-CTLA-4 PE (cat. no.: 349906, clone: L3D10), anti-CD86 APC (cat. no.: 374208, clone: BU63), anti-CD200 PE (cat. no.: 399804, clone: A18042B), and anti-CD200R APC (cat. no.: 329308, clone: OX-108), all sourced from BioLegend (San Diego, CA, USA). For precise gating during cytometric analysis, FMO controls were incorporated for immune checkpoint antibodies. Subsequent to the antibody incubation phase, erythrocytes were lysed using a specific lysing buffer from BD (Franklin Lakes, NJ, USA). The cell suspension post-lysis was then subjected to a wash step and interrogated using a CytoFLEX LX cytometer (Beckman Coulter, Indianapolis, IN, USA). For downstream data processing, Kaluza Analysis v 2.1 software, also from Beckman Coulter (Indianapolis, USA) was used. Routine quality assurance for the CytoFLEX LX system was maintained using CytoFLEX Ready to Use Daily QC Fluorospheres reagents (Beckman Coulter, Indianapolis, USA). Figure 1 and Figure 2 depict the resultant analysis from the samples.

### 2.4. Serological Profiling of Anti-EBV Specific Antibodies

We executed a qualitative analysis to detect the presence of specific IgA, IgM, and IgG antibodies targeted against selected Epstein–Barr virus (EBV) antigens: viral-capsid antigen (VCA), early antigen (EA), and Epstein–Barr nuclear antigen 1 (EBNA1) in the sera of both study and control cohorts. We leveraged commercial ELISA kits designed to discern antibodies of classes IgA, IgG, and IgM specific to these EBV antigens. Protocols followed the stipulated guidelines of the kit manufacturer. The assay used a suite of kits: EBV VCA IgA, EBV EA IgA, EBV EBNA1 IgA, EBV VCA IgG, EBV EA IgG, EBV EBNA1 IgG, EBV VCA IgM, EBV EA IgM, and EBV EBNA1 IgM (Demeditec Diagnostics GmbH, Germany). Absorbance readings were captured using a VictorTM3 microplate reader (PerkinElmer, Waltham, MA, USA). Antibody titers, quantified from these readings, were represented as U/mL, adhering to the calibration guidelines provided by the manufacturer. A titer value surpassing 11 was designated as positive.

### 2.5. Assessment of Soluble Immune Checkpoint and Ligand Concentrations in Serum

The concentration of all tested molecules was evaluated with immunoenzymatic assays using serum samples collected from all participants. Commercial kits were used according to manufacturer instructions:

- Human CD200 ELISA Kit (Sensitivity: 20 pg/mL) from Invitrogen, Waltham, MA, USA;
- Human CD200R ELISA Kit (Sensitivity: 11.89 pg/mL) from Abcam, Cambridge, UK;
- Human CTLA-4 ELISA Kit (Sensitivity: 0.13 ng/mL) from Invitrogen, Waltham, MA, USA;
- Human CD86 ELISA Kit (Sensitivity: 0.82 ng/mL) from Invitrogen, Waltham, MA, USA;
- Human PD-1 ELISA Kit (Sensitivity: 1.14 pg/mL) from Invitrogen, Waltham, MA, USA;
- Human PD-L1 ELISA Kit (Sensitivity: 0.6 pg/mL) from Invitrogen, Waltham, MA, USA.

Measurements were conducted using a VictorTM3 reader (PerkinElmer, Waltham, MA, USA).

### 2.6. Statistical Analysis of Obtained Data

The acquired data underwent statistical analysis utilizing Tibco Statistica 13.3 software, Palo Alto, CA, USA. The distribution normality of data was assessed using the Shapiro–Wilk test. Differences between groups were analyzed using the Kruskal–Wallis test followed by Dunn’s post hoc test. The *p*-values for Dunn’s test were adjusted for multiple comparisons using the Bonferroni method. Spearman’s correlation coefficients were used to explore relationships between pairs of variables. ROC curves were used to assess the diagnostic performance of laboratory tests for patient-related parameters. Data visualizations were generated using GraphPad Prism Software v. 9.4.1, San Diego, CA, USA.

## 3. Results

### 3.1. Classification and Characteristics of Selected Peripheral Blood Parameters of Patients with CLL and CVID in the Context of EBV Reactivation

**Figure 3:**
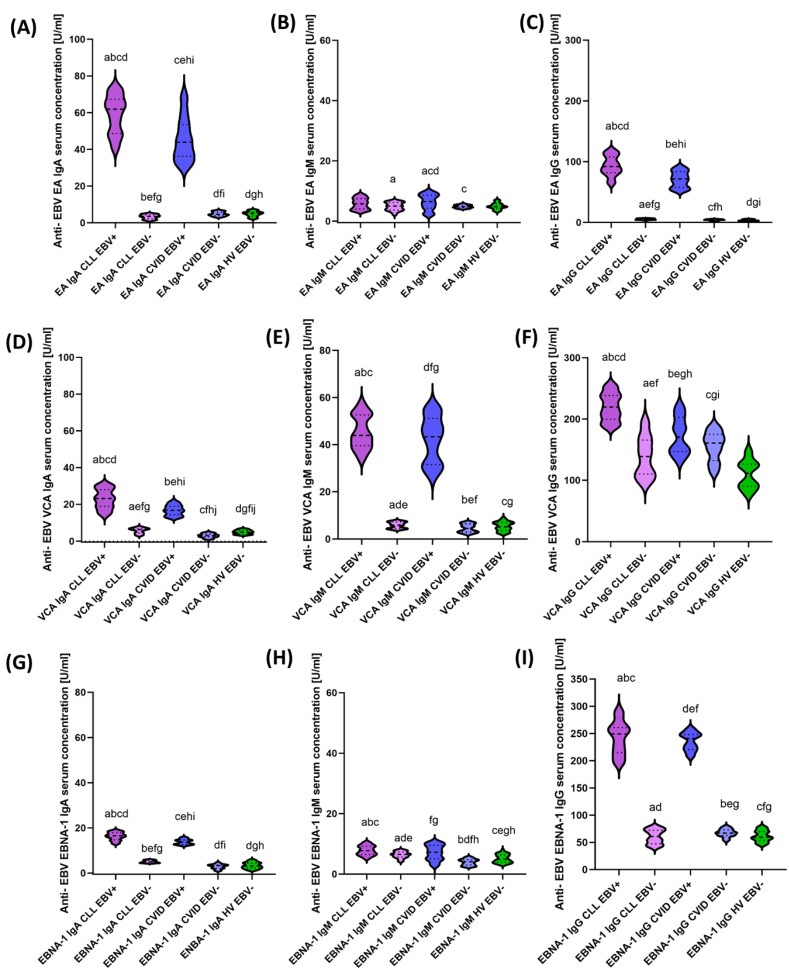
Schematic representation of changes in serological profiles of antibodies against specific EBV antigens in patients with CLL, CVID, and HV, including EBV reactivation. (**A**) Anti−EBV EA IgA serum concentration [U/mL]. (**B**) Anti−EBV EA IgM serum concentration [U/mL]. (**C**) Anti-EBV EA IgG serum concentration [U/mL]. (**D**) Anti−EBV VCA IgA serum concentration [U/mL]. (**E**) Anti−EBV VCA IgM serum concentration [U/mL]. (**F**) Anti−EBV VCA IgG serum concentration [U/mL]. (**G**) Anti-EBV EBNA−-1 IgA serum concentration [U/mL]. (**H**) Anti−EBV EBNA−1 IgM serum concentration [U/mL]. (**I**) Anti−EBV EBNA−1 IgG serum concentration [U/mL]. Letters denote statistically significant results. Individual disease entities are marked with colors, taking into account the reactivation of the EBV virus.

In order to assess selected parameters of immune system functioning, with particular emphasis on the relationship between PD-1/PD-L1, CTLA-4/CD86, and CD200R/CD200 in the context of EBV reactivation, all patients included in this study were tested for the assessment of serological profiles and the number of EBV copies in the tested genetic material, which was the basis for the classification of patients into EBV+ (showing EBV reactivation) and EBV− (patients without reactivation). The analysis of serological profiles of antibodies against specific EBV antigens such as EA (early antigen), VCA (viral-capsid antigen), and EBNA-1 (Epstein–Barr virus nuclear antigen 1) in classes IgA, IgM, and IgG showed EBV reactivation in 60% of patients with CLL and 57.69% of patients with CVID. No EBV reactivation was observed in healthy volunteers enrolled in this study. Detailed antibody serum concentration data and their statistical analyses are presented in Table 1 and Figure 3A–I.

The observed concentration levels of individual antibodies directed against specific EBV antigens were extremely diverse between the individual groups of the analyzed patients. However, all antibody concentrations tested in all classes were higher in EBV+ patients than in EBV− patients, both in CLL and CVID patients. Moreover, the statistical analysis of serological profiles between EBV+ CLL and EBV+ CVID patients showed a statistically significant increase in anti-EBA EA IgA (1.41-fold); anti-EBV EA IgG (1.28-fold); anti-EBV VCA IgA (1.38-fold); anti-EBV VCA IgG (1.29-fold); and anti-EBV EBNA-1 IgA (1.20-fold) in CLL patients compared with CVID (Figure 3A,C,D,F,G). The analysis of the obtained results for this experience of patients in the study group in relation to healthy volunteers is presented in Supplementary Materials Table S1.

**Figure 4:**
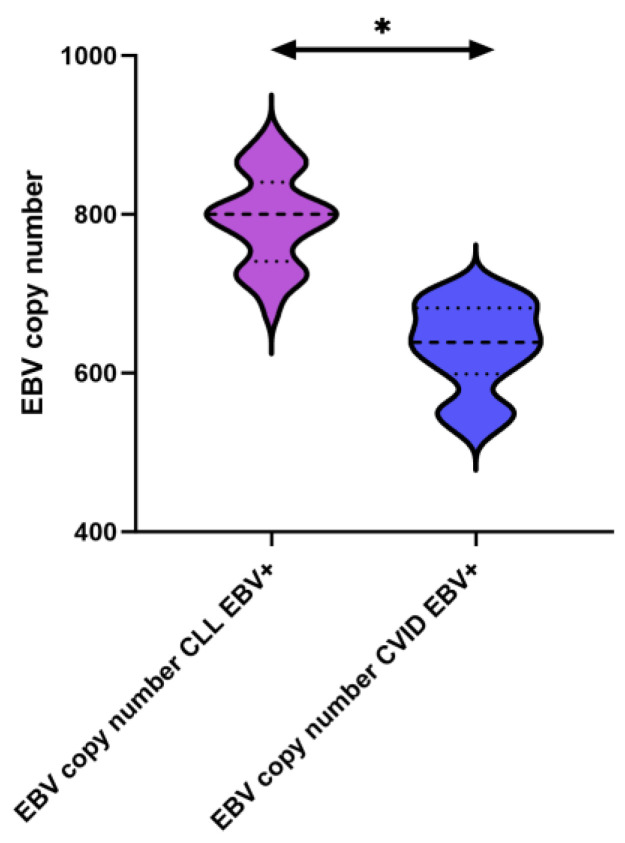
Schematic representation of the assessment of the number of EBV virus copies in the genetic material of patients with CLL, CVID, and HV, including EBV reactivation. * statistically significant results; individual disease entities are marked with colors, taking into account the reactivation of the EBV virus.

The next step was to assess the number of EBV copies in the genetic material, where EBV copies were present only in CLL EBV+ and CVID EBV+ patients. In the remaining cases, the number of EBV copies extracted with the test was below the detection limit of the test used (i.e., below 10 copies). This means that patients below the detection limit of less than 10 copies were labeled as EBV+ patients, while all patients with more than 11 copies were labeled as EBV+. (Table 2). The mean values obtained for patients with CLL EBV+ were 794.21 ± 56.61 virus copies, and for patients with CVID EBV+, the mean values were 633.98 ± 53.69. The analysis of medians obtained for individual groups of patients showed an increase in the number of viral copies in patients with CLL EBV+ relative to CVID EBV+ by more than 25% (Figure 4).

In the next stage of our study, we decided to take a closer look at the selected parameters of peripheral blood, including analyses of morphology, biochemistry, and immunophenotype of patients, taking into account EBV reactivation. In Table 3, we present a comparative analysis of the morphology and biochemistry of peripheral blood from all analyzed patients. As we can see, patients with EBV+ have significantly changed parameters compared with EBV−, both in patients with CLL and CVID. In the case of the first group, we observed an increase in WBC, LYM, MON, and IgM and a decrease in HGB and PLT (Table 3). However, for patients with CVID, we noted a significant decrease in WBC, LYM, MON, HGB, PLT, and IgG (Table 3). The analysis between patients with CLL EBV+ and CVID EBV+ also showed a significant increase in almost all analyzed parameters, except HGB and IgA in patients with CLL in relation to CVID (Table 3). Of course, we also noted a number of significant changes in selected parameters between individual groups of patients and healthy volunteers, which are presented in detail in Supplementary Materials Table S2.

The performed analysis of peripheral blood immunophenotype of all patients also provided extremely valuable information about the state of the immune system for each of the analyzed groups. Of course, some results of peripheral blood immunophenotyping are characteristic of the analyzed disease subunits, such as a higher number of CD19+ B cells in CLL patients and a higher number of TCD3+ cells in CVID patients. The obtained test results and their statistical analysis are presented in Table 4 and Supplementary Materials Table S3 (in relation to healthy volunteers).

EBV reactivation significantly increases the percentage of CD3+ lymphocytes and CD8+ T lymphocytes or CD19+ B lymphocytes in patients diagnosed with CLL. In patients with CVID, no statistically significant changes were observed in the percentage of tested immune cell populations in the context of EBV reactivation (Table 4). However, a comparative analysis of both groups of EBV+ patients with each other showed statistically significant changes in almost every cell population, with the exception of the CD4+/CD8+ ratio.

### 3.2. Analysis of the Effect of EBV Reactivation on the Percentage of Lymphocytes Expressing Positive Immune Checkpoints and Their Ligands

**Figure 5:**
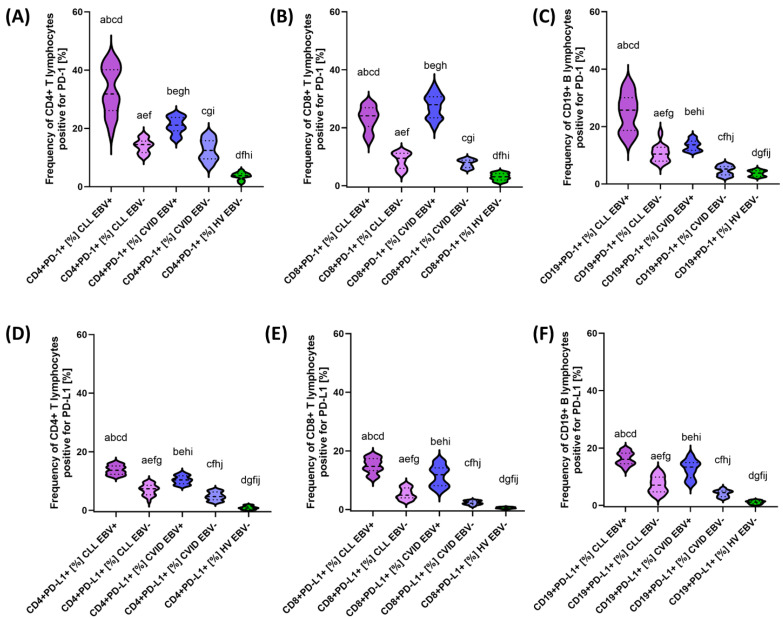
Schematic representation of the percentage of peripheral blood lymphocytes positive for PD−1 and PD−L1 expression in patients with CLL, CVID, and HV, including EBV reactivation. (**A**) Frequency of CD4+ T lymphocytes positive for PD−1 [%]. (**B**) Frequency of CD8+ T lymphocytes positive for PD−1 [%]. (**C**) Frequency of CD19+ B lymphocytes positive for PD−1 [%]. (**D**) Frequency of CD4+ T lymphocytes positive for PD−L1 [%]. (**E**) Frequency of CD8+ T lymphocytes positive for PD−L1 [%]. (**F**) Frequency of CD19+ B lymphocytes positive for PD−L1 [%]. Letter designations refer to statistically significant results. Individual disease entities, including EBV reactivation, are marked with different colors.

**Figure 6:**
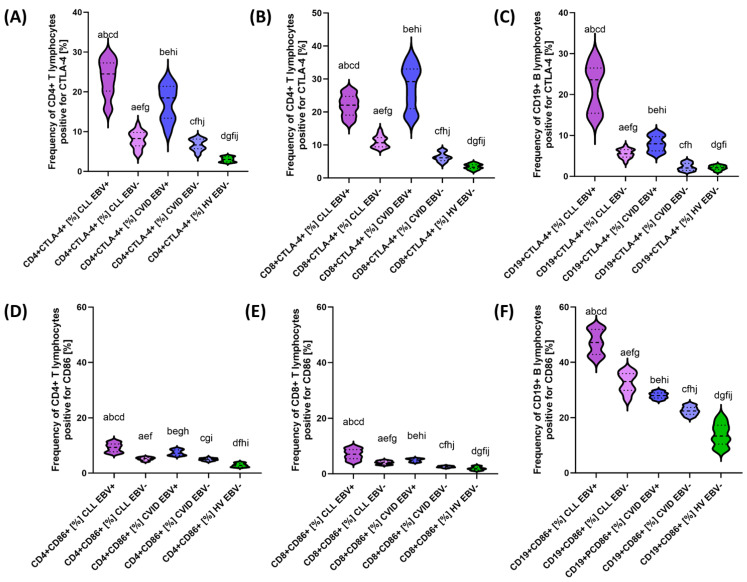
Schematic representation of the percentage of peripheral blood lymphocytes positive for CTLA−4 and CD86 expression in patients with CLL, CVID, and HV, including EBV reactivation. (**A**) Frequency of CD4+ T lymphocytes positive for CTLA-4 [%]. (**B**) Frequency of CD8+ T lymphocytes positive for CTLA−-4 [%]. (**C**) Frequency of CD19+ B lymphocytes positive for CTLA−4 [%]. (**D**) Frequency of CD4+ T lymphocytes positive for CD86 [%]. (**E**) Frequency of CD8+ T lymphocytes positive for CD86 [%]. (**F**) Frequency of CD19+ B lymphocytes positive for CD86 [%]. Letter designations refer to statistically significant results. Individual disease entities, including EBV reactivation, are marked with different colors.

**Figure 7:**
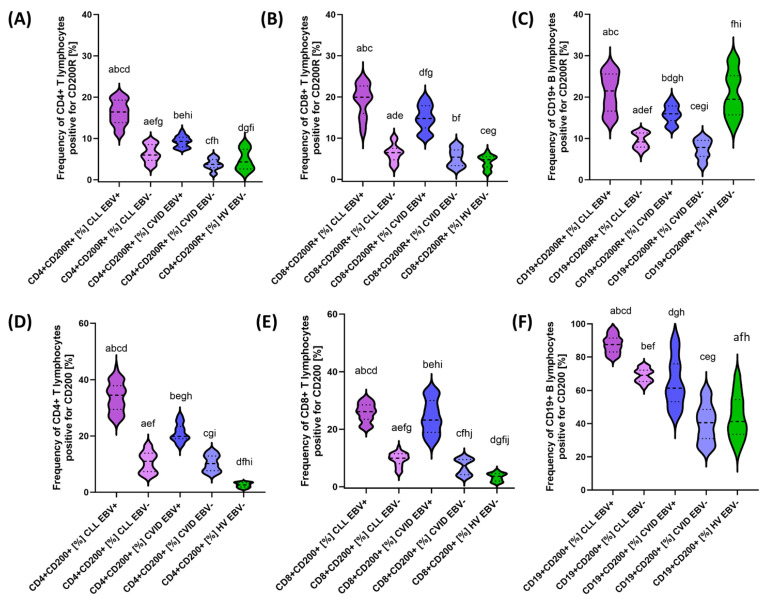
Schematic representation of the percentage of peripheral blood lymphocytes positive for CD200R and CD200 expression in patients with CLL, CVID, and HV, including EBV reactivation. (**A**) Frequency of CD4+ T lymphocytes positive for CD200R [%]. (**B**) Frequency of CD8+ T lymphocytes positive for CD200R [%]. (**C**) Frequency of CD19+ B lymphocytes positive for CD200R [%]. (**D**) Frequency of CD4+ T lymphocytes positive for CD200 [%]. (**E**) Frequency of CD8+ T lymphocytes positive for CD200 [%]. (**F**) Frequency of CD19+ B lymphocytes positive for CD200 [%]. Letter designations refer to statistically significant results. Individual disease entities, including EBV reactivation, are marked with different colors.

After a detailed characterization of the basic parameters of the patients included in this study, we decided to take a closer look at the impact of EBV reactivation on the percentage of selected T and B lymphocyte subpopulations positive for PD-1/PD-L1, CTLA-4/CD86, and CD200R/CD200. The test data obtained are presented collectively in Table 5 and graphically for individual molecules in Figure 5, Figure 6 and Figure 7.

Analysis of EBV reactivation in CLL patients showed a significant increase in the percentage of all analyzed checkpoints and their ligands in all T and B lymphocyte subpopulations compared with EBV− patients (Table 5). In the case of PD-1, the increase was correspondingly higher by 2.20 times for CD4+; 2.55-fold for CD8+; and 2.47-fold for CD19+ (Figure 5A–C), while PD-L1 was 1.86-fold for CD4+, respectively; and 3.00-fold for CD8+ and 2.28-fold for CD19+ (Figure 5D–F). Consecutive changes in CTLA-4 expression were: 2.97-fold for CD4+; 2.07-fold for CD8+; and 2.96-fold for CD19+ (Figure 6A–C), and for CD86: 1.77-fold for CD4+; 1.72-fold for CD8+; and 1.42-fold for CD19+ (Figure 6D–F). For CD200R, these values were 2.74 times, 3.07-fold, and 2.34-fold higher for CD4+, CD8+, and CD19+, respectively (Figure 7A–C); and for CD200: 3.13-fold for CD4+; 2.62-fold for CD8+; and 1.26-fold for CD19+ (Figure 5D–F). These changes emphasize that EBV reactivation significantly increases the expression of the tested immunological checkpoints and their ligands, which may affect the deregulation of the functioning of the immune system.

Similar changes were observed in patients with CVID showing EBV reactivation. The following changes were noted for this group of patients: 1.69-fold CD4+ PD-1+; 3.48-fold CD8+ PD-1+; 2.62-fold CD19+ PD-1+ (Figure 4A–C); 2.22-fold CD4+ PD-L1+; 5.58-fold CD8+ PD-L1+; and 3.01-fold CD19+ PD-L1+ (Figure 5D–F). For the values observed for CTLA-4: 2.77-fold for CD4+; 4.77-fold for CD8+; and 4.28-fold for CD19+ (Figure 6A–C), and for CD86: 1.51-fold for CD4+; 1.97-fold for CD8+; and 1.25-fold for CD19+ (Figure 6D–F). For the CD200R/CD200 pathway, these changes were: 2.49-fold CD4+ CD200R; 2.74 times CD8+ CD200R+; and 2.04 times CD19+ CD200R+ (Figure 7A–C) and 1.95 times CD4+ CD200+; 3.17-fold for CD8+ CD200+; and 1.51-fold for CD19+ CD200+ (Figure 7D–F).

Comparative analysis of patients with CLL EBV+ and CVID EBV+ also showed a number of significant statistical changes in almost all analyzed percentages of T and B lymphocytes positively expressing all tested immunological checkpoints and their ligands, with the exception of CD8+ CD200R+ (Table 5). Moreover, almost most of the analyzed parameters were higher in patients with CLL EBV+, with the exception of CD8+ PD-1+ and CD8+ CTLA-4+, which were 1.16 times and 1.32 times higher, respectively, in patients with CVID EBV+.

The analysis of the percentage of T and B lymphocytes positively expressing all tested immunological checkpoints and their ligands in the compared groups of patients in relation to healthy volunteers is presented in Supplementary Materials Table S4.

### 3.3. Analysis of the Effect of EBV Reactivation on Serum Concentrations of Soluble Forms of Immunological Checkpoints and Their Ligands

**Figure 8:**
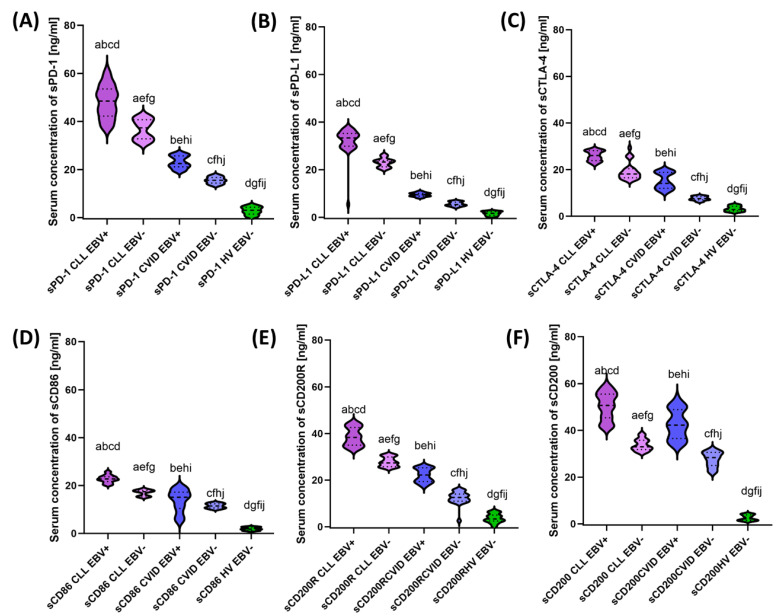
Evaluation of the concentration of soluble forms of the tested molecules in the serum of patients with CLL, CVID, and HV, taking into account EBV reactivation. (**A**) Serum concentration of sPD−1 [ng/mL]. (**B**) Serum concentration of sPD−L1 [ng/mL]. (**C**) Serum concentration of sCTLA−4 [ng/mL]. (**D**) Serum concentration of sCD86 [ng/mL]. (**E**) Serum concentration of sCD200R [ng/mL]. (**F**) Serum concentration of sCD200 [ng/mL]. Statistically significant results are marked with letters. Individual disease entities are marked with colors, taking into account EBV reactivation.

Due to such significant changes observed in the immunophenotypic picture, we also decided to assess the serum concentrations of all tested immunological checkpoints and their ligands in patients included in this study. The test results obtained are presented in Table 6 and Figure 8.

The presented study results confirm the changes observed in the immunophenotype. Here, patients with EBV+ also showed higher serum concentrations of soluble forms of all analyzed molecules in relation to the thigh of EBV− patients in both study populations. In patients with CLL, these changes ranged from 1.30-fold for sPD-1 to 1.54-fold for sCD200, and for patients with CVID, from 1.32-fold for sCD86 to 1.86-fold for sCTLA-4. A detailed analysis of EBV+ patients between both patient populations showed an increase in the concentration of all molecules tested in CLL patients relative to CVID of 1.20-fold for sCD200; 1.50 times for sCD86; 1.73 times for sCD200R; 1.85-fold for sCTLA-4; 2.15 times for sPD-1; and 3.50 for sPD-L1 (Figure 8). The statistical significance observed between the study groups of patients and healthy volunteers are listed in Supplementary Materials Table S5.

### 3.4. Influence of EBV Reactivation on Correlations of Selected Analyzed Parameters of the Immune System

Due to the observed changes, we decided to analyze the effect of EBV reactivation on selected parameters of the immune system of patients with CLL and CVID. The analysis performed is graphically presented in Supplementary Materials Figure S1 for CLL patients and Figure S2 for CVID patients.

Due to the size of the sample, only several dozen statistically significant correlations were recorded in particular groups of patients. For CLL EBV+ patients, we observed 36 significant correlations, of which 10 were negative (four moderate and 6 low) and 26 were positive (seven moderate and 19 low) (Supplementary materials Table S6).

For CLL EBV− patients, there were 25 significant correlations, of which 11 were negative (two high and 9 moderate) and 14 were positive (one high and 13 moderate) (Supplementary materials Table S7).

In CVID EBV+ patients, there were 28 significant correlations, of which 15 were negative (one high, 10 moderate, and four low) and 13 were positive (10 moderate and three low) (Supplementary materials Table S8).

In patients with CVID EBV−, there were 29 significant correlations, of which 14 were negative (one high and 13 moderate) and 15 were positive (one high and 14 moderate) (Supplementary materials Table S9).

### 3.5. Can Changes in Selected Parameters of the Immune System in the Context of EBV Reactivation in Patients with CLL and CVID Be a Potential Diagnostic Marker?

The last stage of the analysis was the assessment of the sensitivity and specificity of the observed changes in the immunophenotype and serological profiles, with particular emphasis on the tested immunological checkpoints and their ligands in the context of EBV reactivation in patients with CLL and CVID.

**Figure 9:**
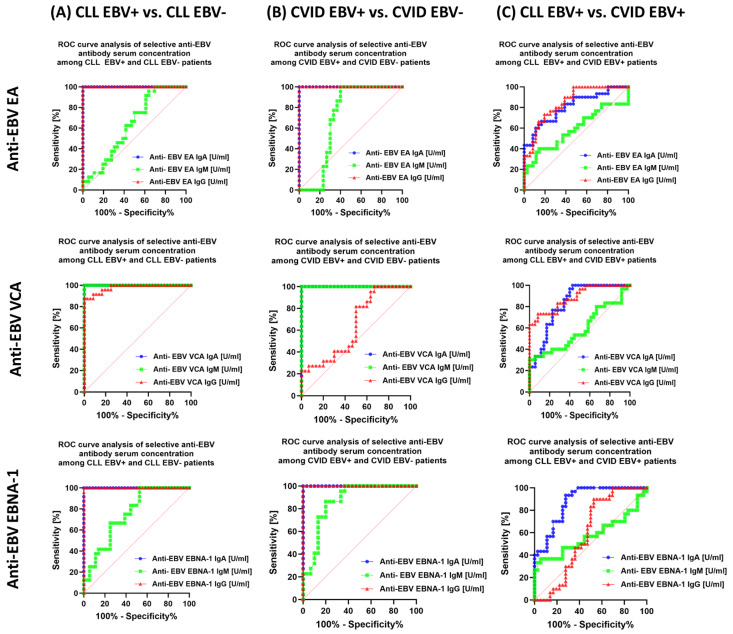
Analysis of ROC curves for the results obtained from analyzing serological profiles of antibodies against specific EBV antigens in the IgA, IgM, and IgG classes in patients with CLL and CVID, including EBV reactivation. (**A**) Analysis of ROC curves for selected anti−EBV EA, anti−EBV VCA, and anti−EBV EBNA−1 antibodies in all three classes of antibodies for EBV+ and EBV− CLL patients. (**B**) Analysis of ROC curves for selected anti−EBV EA, anti−EBV VCA, and anti-EBV EBNA−1 antibodies in all three classes of antibodies for CVID EBV+ and CVID EBV− patients. (**C**) Analysis of ROC curves for selected anti−EBV EA, anti−EBV VCA, and anti−EBV EBNA−1 antibodies in all three classes of antibodies for EBV+ CLL and EBV+ CVID patients.

**Figure 10:**
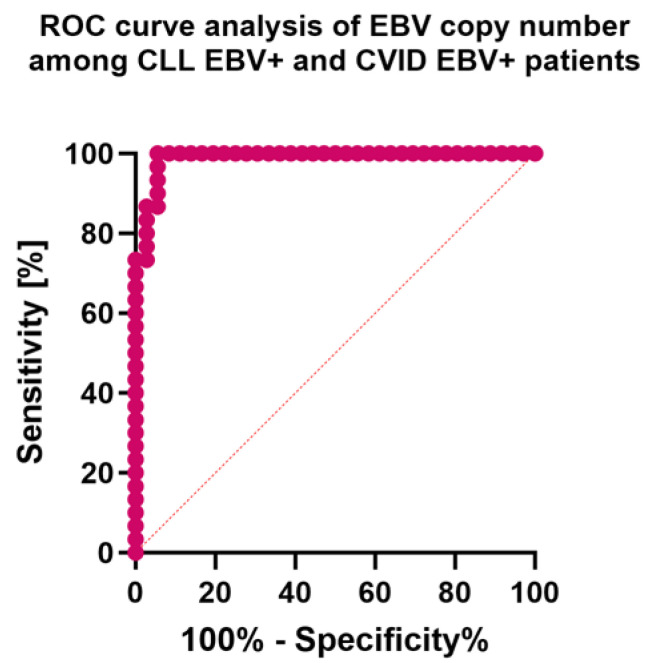
Analysis of ROC curves for the EBV copy number in patients with CLL and CVID, including EBV reactivation.

In the first step, we evaluated the results obtained from analyzing serological profiles of antibodies against specific EBV antigens to confirm their diagnostic effectiveness. These analyses confirmed that for both CLL and CVID patients, highly specific markers of EBV reactivation within the studied populations are: anti-EBV EA IgA and IgG; anti-EBV VCA IgA and IgM; and anti-EBV EBNA-1 IgA and IgG (Figure 9A,B). However, in the case of comparing the results obtained between CLL EBV+ and CVID EBV+ patients, we cannot unambiguously indicate the most sensitive marker indicator to distinguish between these two disease entities (Figure 9C). However, such a function may be performed by assessing the number of EBV virus copies in the examined genetic material (Figure 10).

**Figure 11:**
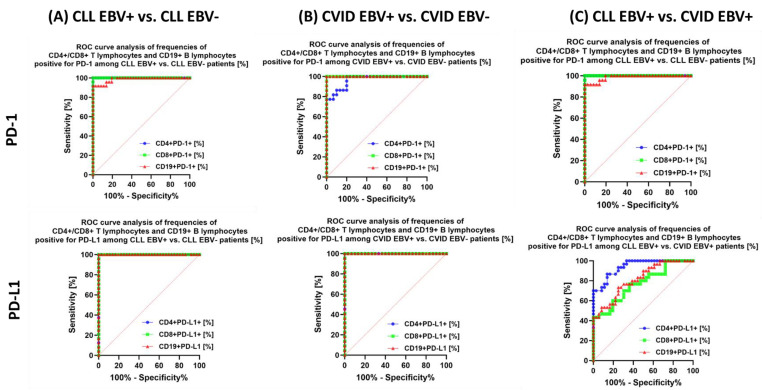
Analysis of ROC curves to assess the percentage of tested T and B lymphocytes showing positive expression of PD−1 and PD−L1 in patients with CLL and CVID, with particular emphasis on EBV reactivation. (**A**) Evaluation of the percentage of tested T and B lymphocytes positive for PD−1 and PD−L1 expression in patients with CLL EBV+ and CLL EBV−. (**B**) Evaluation of the percentage of tested T and B lymphocytes positive for PD−1 and PD−L1 expression in CVID EBV+ and CVID EBV− patients. (**C**) Evaluation of the percentage of tested T and B lymphocytes positive for PD−1 and PD−L1 expression in patients with CLL EBV+ and CVID EBV+.

**Figure 12:**
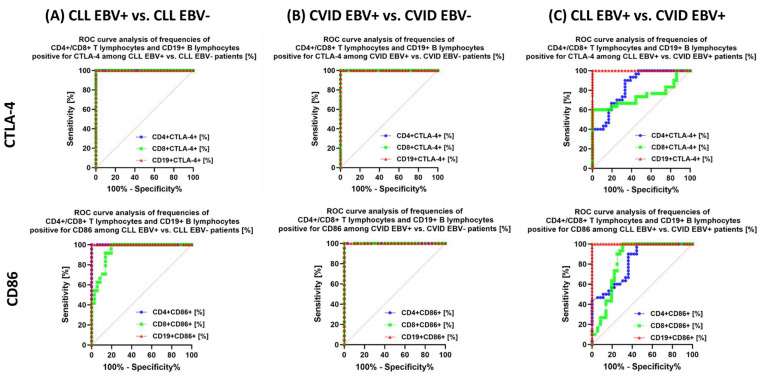
Analysis of ROC curves to assess the percentage of tested T and B lymphocytes showing positive expression of CTLA−4 and CD86 in patients with CLL and CVID, with particular emphasis on EBV reactivation. (**A**) Evaluation of the percentage of tested T and B lymphocytes positive for CTLA−4 and CD86 expression in patients with CLL EBV+ and CLL EBV−. (**B**) Evaluation of the percentage of tested T and B lymphocytes positive for CTLA−4 and CD86 expression in CVID EBV+ and CVID EBV− patients. (**C**) Evaluation of the percentage of tested T and B lymphocytes positive for CTLA-4 and CD86 expression in patients with CLL EBV+ and CVID EBV+.

**Figure 13:**
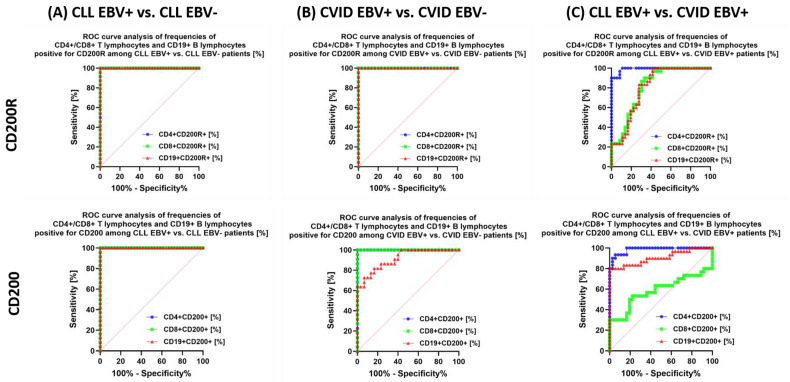
Analysis of ROC curves to assess the percentage of tested T and B lymphocytes showing positive expression of CD200R and CD200 in patients with CLL and CVID, with particular emphasis on EBV reactivation. (**A**) Evaluation of the percentage of tested T and B lymphocytes positive for CD200R and CD200 expression in patients with CLL EBV+ and CLL EBV−. (**B**) Evaluation of the percentage of tested T and B lymphocytes positive for CD200R and CD200 expression in CVID EBV+ and CVID EBV− patients. (**C**) Evaluation of the percentage of tested T and B lymphocytes positive for CD200R and CD200 expression in patients with CLL EBV+ and CVID EBV+.

The analysis also shows that in the case of patients with CLL EBV+ in relation to CLL EBV−, a highly specific marker may be: the percentage of CD4+ PD-1+ and CD8+ PD-1+; percentage of total lymphocytes positive for PD-L1 (Figure 11A) and CTLA-4 (Figure 12A); the percentage of CD4+ CD86+ and CD19+ CD86+ (Figure 12A); and the percentage of total lymphocytes positive for CD200R and CD200 expression (Figure 13A).

**Figure 14:**
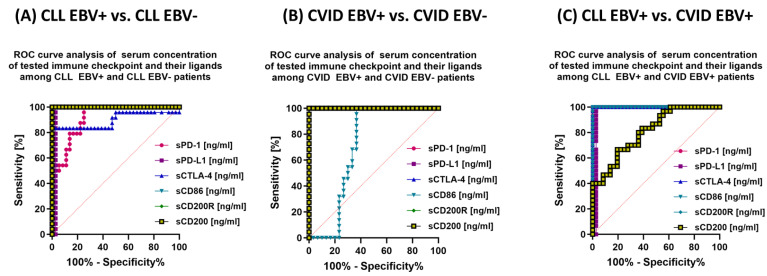
Analysis of ROC curves to assess the serum concentration of soluble forms of the tested immunological checkpoints and their ligands in patients with CLL and CVID, with particular emphasis on EBV reactivation. (**A**) Evaluation of serum concentrations of soluble forms of test immune checkpoints and their ligands in patients with CLL EBV+ and CLL EBV−. (**B**) Evaluation of serum concentrations of soluble forms of test immune checkpoints and their ligands in CVID EBV+ and CVID EBV− patients. (**C**) Evaluation of serum concentrations of soluble forms of the tested immune checkpoints and their ligands in patients with CLL EBV+ and CVID EBV+.

In the case of evaluating the serum concentration of soluble forms of the test molecules, almost all, except for sPD-1 and sCTLA-4, may be potential markers associated with EBV reactivation in this group of patients (Figure 14A).

We observed similar trends in patients with CVID. Of particular note, the potential biomarker molecules of EBV reactivation status are the percentage of CD8 + PD-1 and CD19+ PD-1+ (Figure 11B); the percentage of all tested lymphocytes positive for PD-L1 (Figure 11B), CTLA-4 and CD86 (Figure 12B), and CD200R (Figure 13B) as well as the percentage of CD4+ CD200+ and CD8+ CD200+ (Figure 13B). Serum concentration analysis of the soluble forms of the tested molecules showed that all except sCD86 could be a potential biomarker molecule (Figure 14B).

We recently analyzed the relationship between CLL EBV+ and CVID EBV− patients, which showed a significant advantage of CD4+ PD-1 and CD8+ PD-1 (Figure 11C); CD19+ CTLA-4+ and CD19+ CD86+ (Figure 12C); and all serum concentrations of the molecules tested except for sCD200 (Figure 14C).

Detailed results from this analysis are provided in Supplementary Materials Table S10.

## 4. Discussion

The presented study is a correlational analysis of two important aspects including EBV reactivation and the expression of immune checkpoints and their ligands in the context of two diseases, representing PID-CVID and SID-CLL. The observed significant increase in the expression of immune checkpoints and ligands after EBV reactivation highlights the potential role of this virus in immune dysregulation. The fact that similar changes were observed in both CLL and CVID patients indicates the universality of the effect of EBV on the immune system, regardless of the underlying disease. These findings are consistent with previous studies suggesting a link between EBV and immune modulation, especially in CLL patients. In the work by Gamaleldin et al. from 2021 [44], the researchers showed that high expression of PD-1 and PD-L1 together with high EBD-DNA load was associated with a worse prognosis in CLL. Moreover, studies have shown that B cell lymphomas that are associated with EBV infection express PD-L1, which binds to PD-1, inhibiting T cell cytotoxicity. In the same context, CLL T cells show upregulation of inhibitory molecules, such as PD-1 and PD-L2. In addition, researchers have suggested that PD-1 and PD-L1 may be suitable therapeutic targets for patients suffering from aggressive CLL [44]. This is also confirmed by research by Malpic et al. [45], which showed that CD30 and PD-1/PD-L1 expression appear as potentially unfavorable but targetable biomarkers in EBV-positive diffuse large B-cell lymphoma, and as indicated by the Ozturk study in the course of classical Hodgkin lymphoma [46]. The relationship between EBV reactivation and the PD-1/PD-L1 pathway has been quite well documented in the course of many cancers, including gastric cancer [47,48], breast cancer [49], and nasopharyngeal carcinoma [50], where EBV reactivation was associated with worsening of the tested clinical parameters and a worse prognosis of patients.

The studies conducted by our research team on the interaction between CTLA-4 and CD86 in the course of CLL in the context of EBV reactivation, as well as the currently presented studies, show how important this pathway is in inhibiting the anti-cancer response. The disorders described above are closely related to the presence of EBV genetic material in PBMCs in patients with CLL and the production of antibodies against antigens of this virus, which indicates their involvement in the abnormal cellular response in the course of CLL [51]. The importance of CTLA-4/CD86 is also confirmed in research by other scientists, e.g., work by Mittal et al. [28], Do et al. [29], and Ma et al. [52]. As in the case of PD-1, the CTLA-4 molecule and its participation in the pathogenesis of neoplastic diseases, including EBV reactivation, is quite well documented in the literature, especially in gastric cancer [53,54], breast cancer [55], and epithelial cancers [56].

Studies on the participation of the last CD200R/CD200 pathway analyzed in our work are also scarce. The only study on the relationship between the presence of EBV and the studied pathway in the course of leukemia concerns its immunohistochemical assessment by Christopher Batuello and Emily F Mason from 2023 [57]. Researchers have shown that CD200 can help differentiate Epstein–Barr virus-positive (EBV+) classical Hodgkin lymphoma (CHL) from common Reed-Sternberg cell (RS) and EBV+ large B-cell lymphoma (LBCL).

Very little information can be found on the impact of EBV reactivation and the relationship between immune checkpoints in the course of CVID and its consequences related to the development of neoplastic diseases. Data from the literature indicate that EBV infection in patients with PID may lead to immune dysregulation and increased risk of malignancies, in addition to the severe course of acute infection. Researchers also emphasize that the recognition of various genetic defects and their impact on immune pathways provide us with fundamental insights into the pathophysiology of EBV infection and related diseases and may lead to the development of better-targeted therapies in the future. The studies so far compare EBV infections to several PIDs, which are presented in Table 7.

Comparing CLL and CVID patients with EBV reactivation in this study reveals intriguing differences and similarities in the immune responses of these two patient populations. While both groups show elevated expression of checkpoints and ligands, CLL patients seem to show more pronounced changes. This discrepancy raises questions about potential disease-specific factors that may contribute to changes in immune modulation after EBV reactivation; however, the specific mechanisms require further multifactorial research.

The study of serum concentrations of soluble forms of the analyzed molecules suggests the potential utility of these molecules as biomarkers of EBV reactivation. The distinct patterns observed in CLL and CVID patients, particularly in relation to healthy volunteers, highlight the diagnostic potential of these markers. However, the presented research results concern a small population of patients; therefore, it will be necessary to perform further studies in order to determine the sensitivity and specificity of these markers and their ability to differentiate disease entities for a much larger group of patients.

The present study raises important questions about the functional implications of the observed changes in immune checkpoints and ligands. Increased expression of these molecules may indicate an elevated state of immune activation or exhaustion, potentially affecting immune surveillance, infection response, and disease progression. Future research should aim to elucidate the functional consequences of these changes and their implications for patient outcomes. The marked changes in immune checkpoints and ligands suggest that targeting these pathways may hold promise as a therapeutic approach in CLL and CVID patients with EBV reactivation. Strategies that modulate these checkpoints have the potential to rebalance the immune system and enhance antiviral responses. However, careful consideration of the broader immune landscape and potential off-target effects is critical when designing such interventions.

The studies presented in this publication have some limitations and show only a small part of the changes that occur during the reactivation of the EBV virus. This study’s focus on specific molecules and pathways may not capture the full complexity of immune dysregulation after EBV reactivation, and the observed changes may be incomplete due to sample size. Future research should explore broader immunological profiling to gain a fuller understanding of the changes that occur in CLL and CVID patients and to monitor changes in their bodies over a longer period of time.

Taken together, the analysis of immune checkpoints and ligands in the context of EBV reactivation in CLL and CVID patients sheds light on the intricate relationship between viral infection and immune dysregulation. The findings provide valuable insights into potential diagnostic markers, therapeutic targets, and broader implications of immune modulation in these patient populations. However, further research is needed to uncover the underlying mechanisms, functional consequences, and clinical applications of these observed changes.

## 5. Conclusions

In conclusion, the analysis of EBV reactivation in patients with chronic lymphocytic leukemia (CLL) and common variable immunodeficiency (CVID) revealed substantial alterations in the expression of immunological checkpoints and their ligands across various T and B lymphocyte subpopulations. This investigation underscores a significant increase in the percentages of these checkpoints and ligands in both patient groups compared with EBV-negative individuals. Specifically, the PD-1, PD-L1, CTLA-4, CD86, CD200R, and CD200 pathways exhibited pronounced upregulation in response to EBV reactivation. Notably, the observed changes were more prominent in CLL patients with EBV reactivation compared with those in CVID patients with the same condition. These findings suggest that EBV reactivation plays a crucial role in modifying the immune landscape, potentially contributing to immune dysregulation in both CLL and CVID patients. Furthermore, this study’s serological analysis demonstrated elevated serum concentrations of soluble forms of the analyzed molecules in EBV-positive patients, further affirming the impact of EBV reactivation on immune system modulation. The diagnostic potential of several markers was assessed, revealing distinct patterns that could aid in differentiating between CLL and CVID patients with EBV reactivation. In essence, this correlation analysis highlights the intricate interplay between EBV reactivation and the expression of immunological checkpoints and their ligands in CLL and CVID patients. These findings offer valuable insights into the potential mechanisms underlying immune dysfunction in these conditions and emphasize the significance of further research to elucidate the precise implications of these alterations for patient prognosis and therapeutic interventions.

## Figures and Tables

**Table 1:** Detailed specific anti-EBV antibody serum concentration.

Antibody Serum Concentration [U/mL]		CLL				CVID				HV		*p*-Value	*p*-Value					
		EBV+ (Group 1)		EBV− (Group 2)		EBV+ (Group 3)		EBV+ (Group 4)		EBV− (Group 5)								
		Mean ± SD	Median (Range)	Mean ± SD	Median (Range)	Mean ± SD	Median (Range)	Mean ± SD	Median (Range)	Mean ± SD	Median (Range)		1 vs. 2	1 vs. 3	1 vs. 4	2 vs. 3	3 vs. 4	2 vs. 4
Anti-EBV EA	IgA	59.10 ± 10.19	61.86 (40.21–73.83)	3.30 ± 1.19	3.50 (1.21–4.96)	45.52 ± 11.51	43.89 (30.98–69.49)	4.82 ± 1.11	4.61 (3.07–6.70)	4.84± 1.41	5.10 (2.29–6.96)	0.000 *	0.000 *	0.000 *	0.000 *	0.000 *	0.000 *	0.000 *
	IgM	5.77 ±1.81	5.78 (3.15–8.72)	4.87 ± 1.30	5.02 (2.21–6.77)	6.35 ± 2.48	6.49 (2.06–9.74)	4.86 ± 0.51	4.75 (4.06–5.85)	4.96± 1.03	4.82 (3.15–6.98)	0.000 *	0.086	0.240	0.122	0.017 *	0.015 *	0.973
	IgG	93.32 ± 15.98	91.95 (63.77–119.75)	5.24 ± 1.11	5.07 (3.09–6.97)	70.82 ± 12.88	71.62 (52.58–91.21)	4.26 ± 1.05	4.32 (2.20–5.95)	3.72± 1.20	3.35 (2.16–5.96)	0.000 *	0.000 *	0.000 *	0.000 *	0.000 *	0.000 *	0.001 *
Anti-EBV VCA	IgA	22.98 ± 5.23	23.11 (13.84–31.13)	5.75 ± 1.48	6.34 (3.03–7.88)	16.71± 2.77	16.69 (12.50–21.93)	3.01 ± 1.08	3.04 (1.05–4.91)	4.93 ± 1.08	4.92 (3.07–6.80)	0.000 *	0.000 *	0.000 *	0.000 *	0.000 *	0.000 *	0.000 *
	IgM	45.46 ± 6.98	43.87 (33.73–58.09)	5.89 ± 1.29	5.95 (4.11–7.59)	42.19 ± 9.59	43.31 (26.01–56.58)	4.58 ± 1.67	4.48 (2.22–696)	5.33 ± 1.88	5.19 (2.19–8.83)	0.000 *	0.000 *	0.215	0.000 *	0.000 *	0.000 *	0.005 *
	IgG	219.64 ± 22.14	219.51 (185.99–255.95)	139.19 ± 32.30	138.74 (94.93–199.21)	172.62 ± 29.08	170.33 (130.57–222.41)	155.22 ± 23.27	160.77 (113.07–189.16)	109.50 ± 22.37	111.28 (75.07–151.11)	0.000 *	0.000 *	0.000 *	0.000 *	0.000 *	0.007 *	0.065
Anti-EBV EBNA-1	IgA	16.30 ± 1.70	16.51 (13.01–18.77)	5.04 ± 0.55	4.95 (4.33–5.95)	13.81 ± 1.15	13.79 (12.03–15.87)	2.90 ± 0.99	3.25 (1.02–4.62)	3.36 ± 1.33	3.20 (1.22–5.77)	0.000 *	0.000 *	0.000 *	0.000 *	0.000 *	0.000 *	0.000 *
	IgM	7.84 ± 1.54	7.76 (5.13–10.54)	6.41 ± 1.05	6.53 (4.21–7.98)	7.13 ± 2.45	7.27 (2.73–10.63)	3.97 ± 1.01	4.12 (2.47–5.66)	5.12 ± 1.37	4.88 (3.14–7.96)	0.000 *	0.000 *	0.290	0.000 *	0.265	0.000 *	0.000 *
	IgG	242.84 ± 29.34	249.15 (194.57–294.76)	60.66 ± 12.75	60.91 (41.42–78.80)	235.62 ± 16.08	240.70 (207.77–259.26)	67.27 ± 6.98	67.26 (52.78–79.56)	61.46 ± 9.94	59.40 (45.62–78.50)	0.000 *	0.000 *	0.197	0.000 *	0.000 *	0.000 *	0.132

* statistically significant results.

**Table 2:** Evaluation of the number of EBV virus copies in the genetic material of patients with CLL, CVID, and HV, including EBV reactivation.

	CLL				CVID				HV		*p*-Value
	EBV+		EBV−		EBV+		EBV−		EBV−		
	Mean ± SD	Median (Range)	Mean ± SD	Median (Range)	Mean ± SD	Median (Range)	Mean ± SD	Median (Range)	Mean ± SD	Median (Range)	
EBV copy numer	794.21 ± 56.61	800.24 (676.35–898.53)	N/A	N/A	633.98 ± 53.69	638.89 (528.93–710.49)	N/A	N/A	N/A	N/A	0.000 *

N/A—not applicable; * statistically significant results.

**Table 3:** Characteristics of selected peripheral blood parameters of patients, including EBV reactivation.

Parameter	CLL				CVID				HV		*p*-Value	*p*-Value					
	EBV+ (Group 1)		EBV− (Group 2)		EBV+ (Group 3)		EBV− (Group 4)		EBV− (Group 5)								
	Mean ± SD	Median (Range)	Mean ± SD	Median (Range)	Mean ± SD	Median (Range)	Mean ± SD	Median (Range)	Mean ± SD	Median (Range)		1 vs. 2	1 vs. 3	1 vs. 4	2 vs. 3	3 vs. 4	2 vs. 4
WBC	28.38 ± 4.22	28.57 (20.50–36.21)	26.00 ± 2.95	25.07 (22.23–32.38)	5.94 ± 0.53	6.00 (5.04–6.98)	6.44 ± 0.79	6.20 (5.37–7.73)	5.02 ± 0.43	4.97 (4.28–5.82)	0.000 *	0.032 *	0.000 *	0.000 *	0.000 *	0.003 *	0.000 *
LYM	27.33 ± 4.96	27.65 (19.35–36.21)	19.91 ± 7.23	21.10 (5.88–29.09)	1.18 ± 0.73	1.01 (0.09–2.88)	1.56 ± 0.69	1.53 (0.33–2.86)	2.09 ± 0.50	2.14 (1.03–2.94)	0.000 *	0.000 *	0.000 *	0.000 *	0.000 *	0.003 *	0.000 *
MON	1.16 ± 0.59	1.24 (0.02–1.97)	0.54 ± 0.29	0.52 (0.10–0.98)	0.51 ± 0.29	0.50 (0.01–0.98)	0.83 ± 0.43	0.90 (0.01–1.54)	0.63 ± 0.25	0.65 (0.14–0.95)	0.000 *	0.000 *	0.000 *	0.026 *	0.820	0.005 *	0.000 *
NEU	2.51 ± 0.87	2.48 (1.06–3.93)	2.24 ± 0.88	2.06 (1.12–385)	0.96 ± 0.60	0.91 (0.03–2.00)	1.02 ± 0.60	0.97 (0.03–1.97)	2.63 ± 0.99	2.49 (1.10–3.99)	0.000 *	0.204	0.000 *	0.000 *	0.000 *	0.761	0.000 *
RBC	3.22 ± 0.54	3.44 (2.01–3.98)	3.44 ± 0.88	3.37 (20.6–4.94)	2.89 ± 0.54	2.92 (2.06–3.97)	3.07 ± 0.59	3.18 (1.67–3.99)	4.78 ± 0.90	4.91 (3.10–6.02)	0.000 *	0.422	0.000 *	0.368	0.02 *	0.180	0.190
HGB	9.08 ± 1.33	9.27 (7.01–10.99)	10.49 ± 1.46	10.30 (8.06–12.72)	9.06 ± 0.59	9.02 (8.07–9.98)	10.15 ± 1.17	10.53 (8.25–11.77)	13.96 ± 1.42	14.44 (11.08–15.95)	0.000 *	0.001 *	0.822	0.000 *	0.000 *	0.001 *	0.376
PLT	130.36 ± 11.21	131.04 (111.68–147.84)	159.31 ± 22.24	166.06 (121.23–187.19)	108.70 ± 12.03	109.15 (87.00–127.39)	134.57 ± 7.86	131.91 (123.49–147.92)	280.04 ± 69.73	304.53 (143.11–378.16)	0.000 *	0.000 *	0.000 *	0.204	0.000 *	0.000 *	0.000 *
IgG	6.01 ± 1.72	6.22 (3.10–8.80)	5.87 ± 1.10	5.76 (4.10–7.97)	2.42 ± 0.88	2.23 (1.08–3.80)	3.72 ± 1.15	3.49 (2.09–5.87)	11.49 ± 2.66	11.41 (7.24–15.69)	0.000 *	0.770	0.000 *	0.000 *	0.000 *	0.000 *	0.000 *
IgM	2.03 ± 1.22	1.91 (0.10–3.95)	0.96 ± 0.63	0.95 (0.01–1.99)	1.13 ± 0.49	1.07 (0.18–1.94)	1.04 ± 0.57	1.12 (0.25–1.95)	2.11 ± 0.57	2.02 (1.12–2.95)	0.000 *	0.001 *	0.000 *	0.000 *	0.303	0.490	0.670
IgA	0.48 ± 0.23	0.50 (0.09–0.98)	0.48 ± 0.25	0.50 (0.06–0.83)	0.58 ± 0.25	0.54 (0.06–1.00)	0.52 ± 0.31	0.56 (0.02–0.96)	2.85 ± 0.95	3.19 (1.02–3.97)	0.000 *	0.875	0.140	0.650	0.208	0.587	0.578

* statistically significant results.

**Table 4:** Peripheral blood immunophenotype assessment of patients, including EBV reactivation.

Parameter	CLL				CVID				HV		*p*-Value	*p*-Value					
	EBV+ (Group 1)		EBV− (Group 2)		EBV+ (Group 3)		EBV− (Group 4)		EBV− (Group 5)								
	Mean ± SD	Median (Range)	Mean ± SD	Median (Range)	Mean ± SD	Median (Range)	Mean ± SD	Median (Range)	Mean ± SD	Median (Range)		1 vs. 2	1 vs. 3	1 vs. 4	2 vs. 3	3 vs. 4	2 vs. 4
CD45+ [%]	91.08 ± 4.31	91.35 (82.29–97.33)	93.09 ± 3.05	92.02 (88.50–97.84)	88.31 ± 4.93	87.35 (80.33–97.12)	88.75 ± 4.73	88.70 (80.16–96.73)	93.97 ± 2.35	94.38 (90.38–97.91)	0.000 *	0.100	0.000 *	0.079	0.000 *	0.692	0.000 *
CD3+ [%]	23.52 ± 8.29	23.56 (10.73–37.76)	18.32 ± 4.45	19.47 (10.11–25.07)	65.45 ± 14.83	65.27 (44.27–88.20)	62.42 ± 8.84	61.26 (46.42–77.87)	78.18 ± 8.94	75.16 (65.42–91.94)	0.000 *	0.03 *	0.000 *	0.000 *	0.001 *	0.514	0.000 *
CD19+ [%]	68.34 ± 10.56	70.88 (43.30–86.98)	62.08 ± 8.02	60.43 (46.23–75.09)	9.86 ± 2.90	9.76 (4.25–14.64)	9.43 ± 4.04	9.50 (3.37–16.30)	10.92 ± 2.92	10.29 (7.05–15.88)	0.000 *	0.052 *	0.000 *	0.000 *	0.000 *	0.062	0.000 *
CD4+ [%]	12.55 ± 5.10	11.68 (5.45–21.92)	9.61 ± 3.64	10.38 (3.31–16.70)	32.05 ± 11.35	33.52 (12.03–52.49)	26.73 ± 8.76	24.73 (13.25–39.31)	49.93 ± 5.63	48.69 (40.00–59.89)	0.000 *	0.524	0.000 *	0.000 *	0.000 *	0.847	0.000 *
CD8+ [%]	12.07 ± 6.64	11.13 (2.19–24.81)	10.33 ± 4.26	9.64 (4.06–19.72)	33.41 ± 17.19	33.16 (4.65–69.74)	33.93 ± 13.27	34.91 (10.44–55.17)	32.37 ± 9.86	30.50 (14.26–47.33)	0.000 *	0.011 *	0.000 *	0.000 *	0.000 *	0.720	0.000 *
CD4+/CD8+ ratio	1.66 ± 1.63	1.06 (0.28–8.75)	1.09 ± 0.55	1.13 (0.24–2.26)	1.69 ± 0.89	0.99 (0.23–8.91)	1.03 ± 0.78	0.80 (0.24–2.98)	1.73 ± 0.67	1.58 (0.95–3.51)	0.0028 *	0.380	0.715	0.139	0.829	0.333	0.289

* statistically significant results.

**Table 5:** Evaluation of the percentage of T and B lymphocytes positively expressing the tested immunological checkpoints and their ligands in patients with CLL, CVID, and HV, taking into account EBV reactivation.

Parameter		CLL				CVID				HV		*p*-Value	*p*-Value					
		EBV+ (Group 1)		EBV− (Group 2)		EBV+ (Group 3)		EBV− (Group 4)		EBV− (Group 5)								
		Mean ± SD	Median (Range)	Mean ± SD	Median (Range)	Mean ± SD	Median (Range)	Mean ± SD	Median (Range)	Mean ± SD	Median (Range)		1 vs. 2	1 vs. 3	1 vs. 4	2 vs. 3	3 vs. 4	2 vs. 4
**PD-1**	CD4+ PD-1+	33.02 ± 7.88	31.83 (18.35–44.70)	14.04 ± 2.32	14.46 (10.06–18.11)	21.08 ± 2.80	21.12 (16.07–25.00)	12.62 ± 3.59	12.47 (6.96–18.22)	3.65 ± 1.22	3.80 (1.04–5.71)	0.000 *	0.000 *	0.000 *	0.000 *	0.000 *	0.000 *	0.171
	CD8+ PD-1+	23.23 ± 3.99	24.12 (15.15–28.78)	8.67 ± 2.69	9.45 (4.09–11.99)	27.43 ± 4.03	27.96 (20.25–34.69)	7.67 ± 1.34	8.03 (5.54–9.55)	3.04 ± 1.20	3.05 (1.01–4.88)	0.000 *	0.000 *	0.000 *	0.000 *	0.000 *	0.000 *	0.133
	CD19+ PD-1+	24.85 ± 6.38	25.73 (14.15–35.63)	10.63 ± 2.96	10.42 (6.38–18.43)	13.45 ± 1.78	13.64 (1.01–16.74)	4.67 ± 1.63	5.21 (2.29–6.98)	3.69 ± 1.00	379 (2.08–5.46)	0.000 *	0.000 *	0.000 *	0.000 *	0.000 *	0.000 *	0.000 *
**PD-L1**	CD4+ PD-L1+	13.81 ± 1.55	13.69 (11.32–16.62)	7.04 ± 1.84	7.34 (3.05–9.83)	10.37 ± 1.42	10.40 (8.19–12.99)	4.81 ± 1.39	4.68 (2.85–6.99)	0.89 ± 0.55	0.80 (0.11–1.89)	0.000 *	0.000 *	0.000 *	0.000 *	0.000 *	0.000 *	0.000 *
	CD8+ PD-L1+	14.97 ± 2.51	14.73 (10.31–18.77)	5.38 ± 1.91	4.91 (2.70–8.75)	11.51 ± 3.47	11.89 (6.04–16.97)	2.27 ± 0.65	2.13 (1.02–3.28)	0.56 ± 0.24	0.54 (0.11–0.96)	0.000 *	0.000 *	0.000 *	0.000 *	0.000 *	0.000 *	0.000 *
	CD19+ PD-L1+	16.33 ± 2.19	16.07 (12.27–19.86)	7.23 ± 2.83	7.06 (3.01–12.21)	12.66 ± 3.16	13.38 (7.13–17.87)	4.13 ± 1.13	4.45 (2.17–6.06)	1.07 ± 0.55	1.08 (0.12–1.96)	0.000 *	0.000 *	0.000 *	0.000 *	0.000 *	0.000 *	0.000 *
**CTLA-4**	CD4+ CTLA-4+	23.52 ± 4.41	24.49 (16.06–29.43)	7.99 ± 2.08	8.24 (4.18–11.93)	17.19 ± 4.33	18.48 (9.13–24.00)	6.69 ± 1.38	6.66 (4.08–8.82)	3.02 ± 0.62	3.02 (2.19–3.95)	0.000 *	0.000 *	0.000 *	0.000 *	0.000 *	0.000 *	0.019 *
	CD8+ CTLA-4+	22.01 ± 3.15	22.07 (16.33–26.86)	11.00 ± 1.71	10.67 (8.65–14.69)	27.70 ± 6.38	29.19 (18.15–36.56)	6.33 ± 1.28	6.12 (4.17–8.61)	3.40 ± 0.77	3.26 (2.01–4.72)	0.000 *	0.000 *	0.000 *	0.000 *	0.000 *	0.000 *	0.000 *
	CD19+ CTLA-4+	21.85 ± 5.53	23.62 (13.15–29.63)	5.58 ± 1.06	5.52 (3.32–7.58)	7.98 1.80	7.98 (5.03–10.93)	3.31 ± 0.90	3.06 (1.13–3.86)	2.07 ± 0.53	2.09 (1.01–2.95)	0.000 *	0.000 *	0.000 *	0.000 *	0.000 *	0.000 *	0.000 *
**CD86**	CD4+ CD86+	9.25 ± 1.58	9.31 (7.06–11.86)	5.15 ± 0.54	5.27 (4.08–5.94)	7.42 ± 0.97	7.17 (6.10–8.91)	4.91 ± 0.45	4.87 (4.08–5.81)	2.86 ± 0.64	2.76 (2.02–3.97)	0.000 *	0.000 *	0.000 *	0.000 *	0.000 *	0.000 *	0.097
	CD8+ CD86+	7.03 ± 1.83	7.08 (4.02–9.96)	4.04 ± 0.60	4.11 (3.12–4.95)	4.89 ± 0.55	4.97 (4.01–5.71)	2.51 ± 0.27	2.52 (2.01–2.99)	1.91 ± 0.59	1.83 (1.05–3.00)	0.000 *	0.000 *	0.000 *	0.000 *	0.000 *	0.000 *	0.000 *
	CD19+ CD86+	47.19 ± 4.28	47.15 (40.34–52.97)	32.71 ± 3.56	33.11 (26.11–37.81)	28.07 ± 1.12	28.00 (26.25–29.88)	22.52 ± 1.51	22.42 (20.05–24.93)	13.91 ± 3.96	13.36 (8.03–20.89)	0.000 *	0.000 *	0.000 *	0.000 *	0.000 *	0.000 *	0.000 *
**CD200R**	CD4+ CD200R+	16.26 ± 2.88	16.42 (11.09–20.65)	6.36 ± 2.06	5.99 (3.19–9.48)	9.25 ± 1.30	9.29 (7.40–11.98)	3.75 ± 1.29	3.73 (1.28–5.89)	4.80 ± 2.46	4.33 (1.21–8.99)	0.000 *	0.000 *	0.000 *	0.000 *	0.000 *	0.000 *	0.000 *
	CD8+ CD200R+	19.28 ± 3.87	19.96 (11.08–24.95)	6.35 ± 2.10	6.50 (2.79–10.33)	14.94 ± 3.04	14.78 (10.30–19.93)	5.33 ± 2.09	5.40 (2.26–8.81)	4.27 ± 1.58	4.79 (1.35–6.88)	0.000 *	0.000 *	0.230	0.000 *	0.000 *	0.000 *	0.170
	CD19+ CD200R+	21.11 ± 4.52	21.49 (14.12–27.94)	9.42 ± 1.80	9.17 (6.23–11.88)	15.92 ± 2.18	15.97 (12.24–19.96)	7.40 ± 2.26	7.81 (3.16–10.72)	20.71 ± 5.30	19.48 (12.76–29.42)	0.000 *	0.000 *	0.000 *	0.000 *	0.000 *	0.000 *	0.006 *
**CD200**	CD4 + CD200+	33.80 ± 5.16	34.51 (25.12–43.95)	10.71 ± 3.61	11.02 (5.94–16.96)	21.14 ± 3.13	19.85 (16.15–27.23)	10.30 ± 2.79	10.18 (6.24–14.73)	2.58 ± 0.85	2.62 (1.05–3.75)	0.000 *	0.000 *	0.000 *	0.000 *	0.000 *	0.000 *	0.991
	CD8+ CD200+	25.82 ± 3.16	26.13 (20.32–30.91)	9.54 ± 2.30	9.96 (4.59–13.60)	24.33 ± 5.79	23.22 (15.25–33.71)	6.94 ± 2.70	7.33 (3.11–10.59)	3.37 ± 1.38	3.62 (1.01–5.74)	0.000 *	0.000 *	0.000 *	0.000 *	0.000 *	0.000 *	0.001 *
	CD19+ CD200+	87.76 ± 5.26	87.47 (79.29–97.58)	68.98 ± 3.93	69.12 (61.80–74.99)	64.84 ± 14.22	61.33 (44.17–92.93)	40.34 ± 11.03	40.58 (25.36–59.85)	43.95 ± 12.81	41.27 (23.28–69.37)	0.000 *	0.000 *	0.000 *	0.000 *	0.093	0.000 *	0.000 *

* statistically significant results.

**Table 6:** Evaluation of the concentration of soluble forms of the tested molecules in the serum of patients with CLL, CVID, and HV, taking into account EBV reactivation.

Serum Concentration [ng/mL]	CLL				CVID				HV		*p*-Value	*p*-Value					
	EBV+ (Group 1)		EBV− (Group 2)		EBV+ (Group 3)		EBV− (Group 4)		EBV− (Group 5)								
	Mean ± SD	Median (Range)	Mean ± SD	Median (Range)	Mean ± SD	Median (Range)	Mean ± SD	Median (Range)	Mean ± SD	Median (Range)		1 vs. 2	1 vs. 3	1 vs. 4	2 vs. 3	3 vs. 4	2 vs. 4
sPD-1	48.26 ± 6.65	48.50 (37.00–61.30)	36.70 ± 4.14	37.36 (30.72–42.51)	23.11 ± 2.51	22.52 (19.20–26.97)	15.53 ± 1.35	15.51 (13.18–17.94)	2.88 ± 1.60	3.09 (0.11–5.44)	0.000 *	0.000 *	0.000 *	0.000 *	0.000 *	0.000 *	0.000 *
sPD-L1	32.22 ± 5.35	33.38 (5.47–37.64)	23.03 ± 1.81	23.26 (20.05–26.43)	9.52 ± 0.80	9.52 (8.18–10.96)	5.59 ± 0.86	5.36 (4.08–6.94)	1.61 ± 0.83	1.69 (0.17–2.80)	0.000 *	0.000 *	0.000 *	0.000 *	0.000 *	0.000 *	0.000 *
sCTLA-4	25.90 ± 2.11	26.05 (22.14–28.98)	19.23 ± 3.66	18.13 (15.44–29.34)	15.20 ± 3.37	14.11 (10.15–19.96)	7.62 ± 0.82	7.57 (6.13–8.96)	3.19 ± 1.10	2.87 (1.49–5.13)	0.000 *	0.000 *	0.000 *	0.000 *	0.000 *	0.000 *	0.000 *
sCD86	22.78 ± 1.53	22.73 (20.10–25.95)	16.97 ± 1.21	17.42 (15.04–18.60)	13.72 ± 4.52	15.13 (4.94–19.71)	11.49 ± 0.94	11.48 (10.00–12.93)	1.92 ± 0.65	1.82 (1.00–2.91)	0.000 *	0.000 *	0.000 *	0.000 *	0.000 *	0.000 *	0.000 *
sCD200R	38.60 ± 3.82	38.35 (32.74–44.82)	27.84 ± 2.00	27.39 (25.06–30.94)	22.04 ± 2.85	22.21 (17.26–26.05)	12.42 ± 2.80	12.58 (2.57–15.89)	3.69 ± 1.90	3.36 (0.09–6.96)	0.000 *	0.000 *	0.000 *	0.000 *	0.000 *	0.000 *	0.000 *
sCD200	50.50 ± 5.72	50.70 (41.34–58.99)	33.82 ± 2.42	32.99 (30.53–38.65)	42.62 ± 6.11	42.25 (33.40–53.82)	27.62 ± 3.11	28.38 (22.02–31.85)	2.59 ± 1.21	2.14 (1.04–4.43)	0.000 *	0.000 *	0.000 *	0.000 *	0.000 *	0.000 *	0.000 *

* statistically significant results.

**Table 7:** Studies comparing EBV infections to PIDs.

Disease Entity/Disorder	Mechanism	References
X-linked lymphoproliferative disease (XLP-1)	The heightened vulnerability of XLP-1 patients to EBV infection is likely a result of diminished NK cytolytic activity and reduced CD8+ T cell killing. This susceptibility stems from EBV’s strong attraction to B cells, coupled with the compromised ability of T and NK cells to interact with B cells due to a deficiency in the SLAM receptor-associated pathway.	[58,59]
CD27 deficiency	These patients were documented to exhibit symptomatic primary EBV infection or lymphadenopathy during early life, and a subset also experienced persistent EBV viremia. About half of the individuals developed a malignant neoplasm.	[60]
RASGRP1 deficiency	Patients with RASGRP1 deficiency commonly display a clinical profile characterized by recurrent infections, enlarged liver and spleen (hepatosplenomegaly), swollen lymph nodes (lymphadenopathy), EBV-related excessive lymph cell growth, and the emergence of B-cell lymphoma. Additionally, they may also present autoimmune traits like autoimmune hemolytic anemia, thrombocytopenia, and uveitis.	[61,62]
CD70 deficiency	The clinical signs of CD70 deficiency closely mirror those of CD27 deficiency. In both cases, patients universally exhibit EBV viremia, and the majority of them go on to develop EBV-associated lymphoproliferation or B-cell malignancy, alongside conditions like hypogammaglobulinemia and compromised targeted antibody responses.	[63,64]
Deficiency of the actin regulator—coronin 1A	Dysfunctional calcium flux and the buildup of β-actin at the immune synapse lead to heightened T cell apoptosis and a reduction in CD4+ lymphocyte count. In the case of coronin 1A deficiency, individuals experienced profound infections, and five of them went on to develop B-cell lymphoma induced by EBV.	[65,66]
Serine/threonine kinase 4 (STK4) deficiency	The aberrations in the immune system result in autoimmunity, EBV viremia, and the recurrence of sinopulmonary and mucocutaneous infections, predominantly associated with herpes viruses. Additionally, patients also face susceptibility to other infections caused by viruses such as molluscum contagiosum, fungi like candidiasis, and bacteria like staphylococci.	[67,68]
Activated phosphatidylinositide 3-kinase delta (PI3Kδ) syndrome (APDS)	Dysregulated function leads to the overactivity of the Akt-mTOR pathway, prompting an excessive terminal differentiation of effector lymphocytes, compromised cytokine generation, and hindered immunoglobulin class switching in B cells. Around 30% of APDS patients have EBV infection, which significantly raises the likelihood of B-cell lymphoma development (occurring in 20% of APDS patients who are infected with EBV).	[69,70]
Autoimmune lymphoproliferative syndrome (ALPS)	A deficit in the Fas-mediated apoptotic pathway could elevate the susceptibility to EBV-related lymphomas. Nonetheless, it is plausible that over extended periods of virus transmission, one of the mechanisms by which the immune system regulates EBV within the B cell framework involves the process of Fas-mediated cell elimination.	[71]

## Data Availability

All necessary information regarding the preparation of this work is available on written request from the corresponding author.

## Supplementary Materials

The following supporting information can be downloaded at: https://www.mdpi.com/article/10.3390/cancers15194786/s1, Table S1. Statistical analysis of the concentration of antibodies against specific EBV antigens in the serum of patients with CLL and CVID, taking into account EBV reactivation in relation to HV. Table S2. Statistical analysis of selected parameters of the morphology and biochemistry of peripheral blood of patients with CLL and CVID, including EBV reactivation in relation to HV. Table S3. Statistical analysis of selected peripheral blood immunophenotype parameters of patients with CLL and CVID, including EBV reactivation in relation to HV. Table S4. Statistical analysis of the percentage of T and B lymphocytes positively expressing the tested immunological checkpoints and their ligands in patients with CLL and CVID, including EBV reclamation against HV. Table S5. Statistical analysis of serum concentrations of tested immunological checkpoints and their ligands in patients with CLL and CVID, including EBV reclamation against HV. Table S6. Spearman rank correlations for CLL EBV+ patients. Table S7. Spearman rank correlations for CLL EBV− patients. Table S8. Spearman rank correlations for CVID EBV+ patients. Table S9. Spearman rank correlations for CVID EBV− patients. Table S10. ROC curve analysis. Figure S1. Spearman rank correlation analysis of patients with CLL EBV+ (A) and CLL EBV− (B). Figure S2. Spearman rank correlation analysis of patients with CVID EBV+ (A) and CVID EBV− (B).
