# Supplementary material for: Could Immune Checkpoint Disorders and EBV Reactivation Be Connected in the Development of Hematological Malignancies in Immunodeficient Patients?
Source: Cancers (Basel). 2023 Sep 29;15(19):4786. doi: 10.3390/cancers15194786 (PMC10572023; doi:10.3390/cancers15194786)
Supplement: Supplementary file 1 [file cancers-15-04786-s001.zip › cancers-2578420-supplementary.pdf]

Supplementary Materials

Table S1. Statistical analysis of the concentration of antibodies against specific EBV antigens in the serum of patients with CLL and CVID, taking into account EBV reactivation in relation to HV

Antibody serum concentration [U/ml]		CLL				CVID				Healthy volunteer		p-vale	p-value			
		EBV+ (group 1)		EBV- (group 2)		EBV+ (group 3)		EBV- (group 4)		EBV- (group 5)						
		Mean±SD	Median (Range)	Mean±SD	Median (Range)	Mean±SD	Median (Range)	Mean±SD	Median (Range)	Mean±SD	Median (Range)		1 vs. 5	2 vs. 5	3 vs. 5	4 vs. 5
Anti-EBV EA	IgA	59.10± 10.19	61.86 (40.21-73.83)	3.30± 1.19	3.50 (1.21-4.96)	45.52± 11.51	43.89 (30.98-69.49)	4.82± 1.11	4.61 (3.07-6.70)	4.84± 1.41	5.10 (2.29-6.96)	0.000*	0.000*	0.0001*	0.000*	0,775
	IgM	5.77 ±1.81	5.78 (3.15-8.72)	4.87± 1.30	5.02 (2.21-6.77)	6.35± 2.48	6.49 (2.06-9.74)	4.86± 0.51	4.75 (4.06-5.85)	4.96± 1.03	4.82 (3.15-6.98)	0.000*	0.000*	0.869	0.016*	0.639
	IgG	93.32± 15.98	91.95 (63.77-119.75)	5.24± 1.11	5.07 (3.09-6.97)	70.82± 12.88	71.62 (52.58-91.21)	4.26± 1.05	4.32 (2.20-5.95)	3.72± 1.20	3.35 (2.16-5.96)	0.000*	0.000*	0.000*	0.000*	0.118
Anti-EBV VCA	IgA	22.98± 5.23	23.11 (13.84-31.13)	5.75± 1.48	6.34 (3.03-7.88)	16.71± 2.77	16.69 (12.50-21.93)	3.01± 1.08	3.04 (1.05-4.91)	4.93± 1.08	4.92 (3.07-6.80)	0.000*	0.000*	0.002*	0.000*	0.000*
	IgM	45.46± 6.98	43.87 (33.73-58.09)	5.89± 1.29	5.95 (4.11-7.59)	42.19± 9.59	43.31 (26.01-56.58)	4.58± 1.67	4.48 (2.22-696)	5.33± 1.88	5.19 (2.19-8.83)	0.000*	0.000*	0.286	0.000*	0.157
	IgG	219.64± 22.14	219.51 (185.99-255.95)	139.19± 32.30	138.74)94.93-199.21)	172.62± 29.08	170.33 (130.57-222.41)	155.22± 23.27	160.77 (113.07-189.16)	109.50 ±22.37	111.28 (75.07-151.11)	0.000*	0.000*	0.000*	0.000*	0.000*
Anti-EBV EBNA-1	IgA	16.30± 1.70	16.51 (13.01-18.77)	5.04± 0.55	4.95 (4.33-5.95)	13.81± 1.15	13.79 (12.03-15.87)	2.90± 0.99	3.25 (1.02-4.62)	3.36± 1.33	3.20 (1.22-5.77)	0.000*	0.000*	0.000*	0.000*	0.289
	IgM	7.84± 1.54	7.76 (5.13-10.54)	6.41± 1.05	6.53 (4.21-7.98)	7.13± 2.45	7.27 (2.73-10.63)	3.97± 1.01	4.12 (2.47-5.66)	5.12± 1.37	4.88 (3.14-7.96)	0.000*	0.000*	0.000*	0.000*	0.006*
	IgG	242.84 ±29.34	249.15 (194.57-294.76)	60.66± 12.75	60.91 (41.42-78.80)	235.62 ±16.08	240.70 (207.77-259.26)	67.27± 6.98	67.26 (52.78-79.56)	61.46± 9.94	59.40 (45.62-78.50)	0.000*	0.000*	0.762	0.000*	0.039*

Table S2. Statistical analysis of selected parameters of the morphology and biochemistry of peripheral blood of patients with CLL and CVID, including EBV reactivation in relation to HV

Parameter	CLL				CVID				HV		p-value	p-value			
	EBV+ (group 1)		EBV+ (group 2)		EBV+ (group 3)		EBV- (group 4)		EBV- (group 5)						
	Mean±SD	Median (Range)	Mean±SD	Median (Range)	Mean±SD	Median (Range)	Mean±SD	Median (Range)	Mean±SD	Median (Range)		1 vs. 5	2 vs. 5	3 vs. 5	4 vs. 5
**WBC**	28.38± 4.22	28.57 (20.50-36.21)	26.00± 2.95	25.07 (22.23-32.38)	5.94± 0.53	6.00 (5.04-6.98)	6.44± 0.79	6.20 (5.37-7.73)	5.02± 0.43	4.97 (4.28-5.82)	0.000*	0.000*	0.000*	0.000*	0.000*
**LYM**	27.33± 4.96	27.65 (19.35-36.21)	19.91± 7.23	21.10 (5.88-29.09)	1.18± 0.73	1.01 (0.09-2.88)	1.56± 0.69	1.53 (0.33-2.86)	2.09± 0.50	2.14 (1.03-2.94)	0.000*	0.000*	0.000*	0.000*	0,007*
**MON**	1.16± 0.59	1.24 (0.02-1.97)	0.54± 0.29	0.52 (0.10-0.98)	0.51± 0.29	0.50 (0.01-0.98)	0.83± 0.43	0.90 (0.01-1.54)	0.63± 0.25	0.65 (0.14-0.95)	0.000*	0,534	0,294	0,159	0,052
**NEU**	2.51± 0.87	2.48 (1.06-3.93)	2.24± 0.88	2.06 (1.12-385)	0.96± 0.60	0.91 (0.03-2.00)	1.02± 0.60	0.97 (0.03-1.97)	2.63± 0.99	2.49 (1.10-3.99)	0.000*	0,000*	0,119	0,000*	0,000*
**RBC**	3.22± 0.54	3.44 (2.01-3.98)	3.44± 0.88	3.37 (20.6-4.94)	2.89± 0.54	2.92 (2.06-3.97)	3.07± 0.59	3.18 (1.67-3.99)	4.78± 0.90	4.91 (3.10-6.02)	0.000*	0.000*	0.000*	0.000*	0.000*
**HGB**	9.08± 1.33	9.27 (7.01-10.99)	10.49± 1.46	10.30 (8.06-12.72)	9.06± 0.59	9.02 (8.07-9.98)	10.15± 1.17	10.53 (8.25-11.77)	13.96± 1.42	14.44 (11.08-15.95)	0.000*	0.000*	0.000*	0.000*	0.000*
**PLT**	130.36 ±11.21	131.04 (111.68-147.84)	159.31± 22.24	166.06 (121.23-187.19)	108.70± 12.03	109.15 (87.00-127.39)	134.57± 7.86	131.91 (123.49-147.92)	280.04 ±69.73	304.53 (143.11-378.16)	0.000*	0.000*	0.000*	0.000*	0.000*
**IgG**	6.01± 1.72	6.22 (3.10-8.80)	5.87± 1.10	5.76 (4.10-7.97)	2.42± 0.88	2.23 (1.08-3.80)	3.72± 1.15	3.49 (2.09-5.87)	11.49± 2.66	11.41 (7.24-15.69)	0.000*	0.000*	0.000*	0.000*	0.000*
**IgM**	2.03± 1.22	1.91 (0.10-3.95)	0.96± 0.63	0.95 (0.01-1.99)	1.13± 0.49	1.07 (0.18-1.94)	1.04± 0.57	1.12 (0.25-1.95)	2.11± 0.57	2.02 (1.12-2.95)	0.000*	0.595	0.000*	0.000*	0.000*
**IgA**	0.48± 0.23	0.50 (0.09-0.98)	0.48± 0.25	0.50 (0.06-0.83)	0.58± 0.25	0.54 (0.06-1.00)	0.52± 0.31	0.56 (0.02-0.96)	2.85± 0.95	3.19 (1.02-3.97)	0.000*	0.000*	0.000*	0.000*	0.000*

Table S3. Statistical analysis of selected peripheral blood immunophenotype parameters of patients with CLL and CVID, including EBV reactivation in relation to HV

Parameter	Cll				CVID				HV		p-vale	p-value			
	EBV+ (group 1)		EBV- (group 2)		EBV+ (group31)		EBV- (group 4)		EBV- (group 5)						
	Mean±SD	Median (Range)	Mean±SD	Median (Range)	Mean±SD	Median (Range)	Mean±SD	Median (Range)	Mean±SD	Median (Range)		1 vs. 5	2 vs. 5	3 vs. 5	4 vs. 5
**CD45+ [%]**	91.08± 4.31	91.35 (82.29-97.33)	93.09± 3.05	92.02 (88.50-97.84)	88.31± 4.93	87.35 (80.33-97.12)	88.75± 4.73	88.70 (80.16-96.73)	93.97± 2.35	94.38 (90.38-97.91)	0.000*	0.000*	0.256	0.000*	0.000*
**CD3+ [%]**	23.52± 8.29	23.56 (10.73-37.76)	18.32± 4.45	19.47 (10.11-25.07)	65.45± 14.83	65.27 (44.27-88.20)	62.42± 8.84	61.26 (46.42-77.87)	78.18± 8.94	75.16 (65.42-91.94)	0.000*	0.000*	0.000*	0.000*	0.000*
**CD19+ [%]**	68.34± 10.56	70.88 (43.30-86.98)	62.08± 8.02	60.43 (46.23-75.09)	9.86 ±2.90	9.76 (4.25-14.64)	9.43± 4.04	9.50 (3.37-16.30)	10.92± 2.92	10.29 (7.05-15.88)	0.000*	0.000*	0.000*	0.328	0.160
**CD4+ [%]**	12.55± 5.10	11.68 (5.45-21.92)	9.61± 3.64	10.38 (3.31-16.70)	32.05± 11.35	33.52 (12.03-52.49)	26.73± 8.76	24.73 (13.25-39.31)	49.93± 5.63	48.69 (40.00-59.89)	0.000*	0.000*	0.000*	0.000*	0.000*
**CD8+ [%]**	12.07± 6.64	11.13 (2.19-24.81)	10.33± 4.26	9.64 (4.06-19.72)	33.41± 17.19	33.16 (4.65-69.74)	33.93± 13.27	34.91 (10.44-55.17)	32.37± 9.86	30.50 (14.26-47.33)	0.000*	0.000*	0.000*	0.007*	0.490
**CD4+/CD8+ ratio**	1.66± 1.63	1.06 (0.28-8.75)	1.09± 0.55	1.13 (0.24-2.26)	1.69± 0.89	0.99 (0.23-8.91)	1.03± 0.78	0.80 (0.24-2.98)	1.73± 0.67	1.58 (0.95-3.51)	0.0028*	0.053	0.000*	0.020*	0.000*

Table S4. Statistical analysis of the percentage of T and B lymphocytes positively expressing the tested immunological checkpoints and their ligands in patients with CLL and CVID, including EBV reclamation against HV

Parameter		CL1				CVID				HV		p-vale	p-value			
		EBV+ (group 1)		EBV- (group 2)		EBV+ (group3)		EBV+ (group 4)		EBV- (group 5)						
		Mean±SD	Median (Range)	Mean±SD	Median (Range)	Mean±SD	Median (Range)	Mean±SD	Median (Range)	Mean±SD	Median (Range)		1 vs. 5	2 vs. 5	3 vs. 5	4 vs. 5
**PD-1**	**CD4+PD-1+**	33.02± 7.88	31.83 (18.35-44.70)	14.04± 2.32	14.46 (10.06-18.11)	21.08± 2.80	21.12 (16.07-25.00)	12.62± 3.59	12.47 (6.96-18.22)	3.65± 1.22	3.80 (1.04-5.71)	0.000*	0.000*	0.000*	0.000*	0.000*
	**CD8+PD-1+**	23.23± 3.99	24.12 (15.15-28.78)	8.67± 2.69	9.45 (4.09-11.99)	27.43± 4.03	27.96 (20.25-34.69)	7.67± 1.34	8.03 (5.54-9.55)	3.04± 1.20	3.05 (1.01-4.88)	0.000*	0.000*	0.000*	0.000*	0.000*
	**CD19+PD-1+**	24.85± 6.38	25.73 (14.15-35.63)	10.63± 2.96	10.42 (6.38-18.43)	13.45 ±1.78	13.64 (1.01-16.74)	4.67± 1.63	5.21 (2.29-6.98)	3.69± 1.00	379 (2.08-5.46)	0.000*	0.000*	0.000*	0.000*	0.003*
**PD-L1**	**CD4+PD-L1+**	13.81± 1.55	13.69 (11.32-16.62)	7.04± 1.84	7.34 (3.05-9.83)	10.37± 1.42	10.40 (8.19-12.99)	4.81± 1.39	4.68 (2.85-6.99)	0.89± 0.55	0.80 (0.11-1.89)	0.000*	0.000*	0.000*	0.000*	0.000*
	**CD8+ PD-L1+**	14.97± 2.51	14.73 (10.31-18.77)	5.38± 1.91	4.91 (2.70-8.75)	11.51± 3.47	11.89 (6.04-16.97)	2.27± 0.65	2.13 (1.02-3.28)	0.56± 0.24	0.54 (0.11-0.96)	0.000*	0.000*	0.000*	0.000*	0.000*
	**CD19+ PD-L1+**	16.33± 2.19	16.07 (12.27-19.86)	7.23± 2.83	7.06 (3.01-12.21)	12.66± 3.16	13.38 (7.13-17.87)	4.13± 1.13	4.45 (2.17-6.06)	1.07± 0.55	1.08 (0.12-1.96)	0.000*	0.000*	0.000*	0.000*	0.000*
**CTLA-4**	**CD4+ CTLA-4+**	23.52± 4.41	24.49 (16.06-29.43)	7.99± 2.08	8.24 (4.18-11.93)	17.19± 4.33	18.48 (9.13-24.00)	6.69± 1.38	6.66 (4.08-8.82)	3.02± 0.62	3.02 (2.19-3.95)	0.000*	0.000*	0.000*	0.000*	0.000*
	**CD8+ CTLA-4+**	22.01± 3.15	22.07 (16.33-26.86)	11.00± 1.71	10.67 (8.65-14.69)	27.70± 6.38	29.19 (18.15-36.56)	6.33± 1.28	6.12 (4.17-8.61)	3.40± 0.77	3.26 (2.01-4.72)	0.000*	0.000*	0.000*	0.000*	0.000*
	**CD19+ CTLA-4+**	7.98 1.80	7.98 (5.03-10.93)	2.31± 0.90	2.06 (1.13-3.86)	21.85± 5.53	23.62 (13.15-29.63)	5.58± 1.06	5.52 (3.32-7.58)	2.07± 0.53	2.09 (1.01-2.95)	0.000*	0.000*	0.000*	0.000*	0.000*
**CD86**	**CD4+ CD86+**	9.25± 1.58	9.31 (7.06-11.86)	5.15± 0.54	5.27 (4.08-5.94)	7.42± 0.97	7.17 (6.10-8.91)	4.91± 0.45	4.87 (4.08-5.81)	2.86± 0.64	2.76 (2.02-3.97)	0.000*	0.000*	0.000*	0.000*	0.000*
	**CD8+ CD86+**	7.03± 1.83	7.08	4.04± 0.60	4.11	4.89± 0.55	4.97	2.51± 0.27	2.52	1.91± 0.59	1.83	0.000*	0.000*	0.000*	0.000*	0.000*

			(4.02-9.96)		(3.12-4.95)		(4.01-5.71)		(2.01-2.99)		(1.05-3.00)					
	**CD19+ CD86+**	47.19± 4.28	47.15 (40.34-52.97)	32.71± 3.56	33.11 (26.11-37.81)	28.07± 1.12	28.00 (26.25-29.88)	22.52± 1.51	22.42 (20.05-24.93)	13.91± 3.96	13.36 (8.03-20.89)	0.000*	0.000*	0.000*	0.000*	0.000*
**CD200R**	**CD4+CD200R+**	16.26± 2.88	16.42 (11.09-20.65)	6.36± 2.06	5.99 (3.19-9.48)	9.25± 1.30	9.29 (7.40-11.98)	3.75± 1.29	3.73 (1.28-5.89)	4.80± 2.46	4.33 (1.21-8.99)	0.000*	0.000*	0.010*	0.000*	0.315
	**CD8+ CD200R +**	19.28± 3.87	19.96 (11.08-24.95)	6.35± 2.10	6.50 (2.79-10.33)	14.94±3.04	14.78 (10.30-19.93)	5.33± 2.09	5.40 (2.26-8.81)	4.27± 1.58	4.79 (1.35-6.88)	0.000*	0.000*	0.000*	0.000*	0.093
	**CD19+ CD200R +**	21.11± 4.52	21.49 (14.12-27.94)	9.42± 1.80	9.17 (6.23-11.88)	15.92± 2.18	15.97 (12.24-19.96)	7.40± 2.26	7.81 (3.16-10.72)	20.71± 5.30	19.48 (12.76-29.42)	0.000*	0.803	0.000*	0.000*	0.000*
**CD200**	**CD4+CD200+**	33.80± 5.16	34.51 (25.12-43.95)	10.71± 3.61	11.02 (5.94-16.96)	21.14± 3.13	19.85 (16.15-27.23)	10.30± 2.79	10.18 (6.24-14.73)	2.58± 0.85	2.62 (1.05-3.75)	0.000*	0.000*	0.000*	0.000*	0.000*
	**CD8+ CD200+**	25.82± 3.16	26.13 (20.32-30.91)	9.54± 2.30	9.96 (4.59-13.60)	24.33± 5.79	23.22 (15.25-33.71)	6.94± 2.70	7.33 (3.11-10.59)	3.37± 1.38	3.62 (1.01-5.74)	0.000*	0.000*	0.000*	0.000*	0.000*
	**CD19+ CD200+**	87.76± 5.26	87.47 (79.29-97.58)	68.98± 3.93	69.12 (61.80-74.99)	64.84± 14.22	61.33 (44.17-92.93)	40.34± 11.03	40.58 (25.36-59.85)	43.95± 12.81	41.27 (23.28-69.37)	0.000*	0.000*	0.000*	0.000*	0.392

Table S5. Statistical analysis of serum concentrations of tested immunological checkpoints and their ligands of patients with CLL and CVID, including EBV reclamation against HV

Serum concentration [ng/ml]	CLl				CVID				Healthy volunteer		p-vale	p-value			
	EBV+ (group 1)		EBV+ (group 2)		EBV+ (group 3)		EBV- (group 4)		EBV- (group 5)						
	Mean±SD	Median (Range)	Mean±SD	Median (Range)	Mean±SD	Median (Range)	Mean±SD	Median (Range)	Mean±SD	Median (Range)		1 vs. 5	2 vs. 5	3 vs. 5	4 vs. 5
**sPD-1**	48.26± 6.65	48.50 (37.00-61.30)	36.70± 4.14	37.36 (30.72-42.51)	23.11± 2.51	22.52 (19.20-26.97)	15.53± 1.35	15.51 (13.18-17.94)	2.88± 1.60	3.09 (0.11-5.44)	0.000*	0.000*	0.000*	0.000*	0.000*
**sPD-L1**	32.22± 5.35	33.38 (5.47-37.64)	23.03± 1.81	23.26 (20.05-26.43)	9.52± 0.80	9.52 (8.18-10.96)	5.59± 0.86	5.36 (4.08-6.94)	1.61± 0.83	1.69 (0.17-2.80)	0.000*	0.000*	0.000*	0.000*	0.000*
**sCTLA-4**	25.90± 2.11	26.05 (22.14-28.98)	19.23± 3.66	18.13 (15.44-29.34)	15.20± 3.37	14.11 (10.15-19.96)	7.62± 0.82	7.57 (6.13-8.96)	3.19± 1.10	2.87 (1.49-5.13)	0.000*	0.000*	0.000*	0.000*	0.000*
**sCD86**	22.78± 1.53	22.73 (20.10-25.95)	16.97± 1.21	17.42 (15.04-18.60)	13.72± 4.52	15.13 (4.94-19.71)	11.49± 0.94	11.48 (10.00-12.93)	1.92± 0.65	1.82 (1.00-2.91)	0.000*	0.000*	0.000*	0.000*	0.000*
**sCD200R**	38.60± 3.82	38.35 (32.74-44.82)	27.84± 2.00	27.39 (25.06-30.94)	22.04± 2.85	22.21 (17.26-26.05)	12.42± 2.80	12.58 (2.57-15.89)	3.69± 1.90	3.36 (0.09-6.96)	0.000*	0.000*	0.000*	0.000*	0.000*
**sCD200**	50.50± 5.72	50.70 (41.34-58.99)	33.82± 2.42	32.99 (30.53-38.65)	42.62± 6.11	42.25 (33.40-53.82)	27.62± 3.11	28.38 (22.02-31.85)	2.59± 1.21	2.14 (1.04-4.43)	0.000*	0.000*	0.000*	0.000*	0.000*

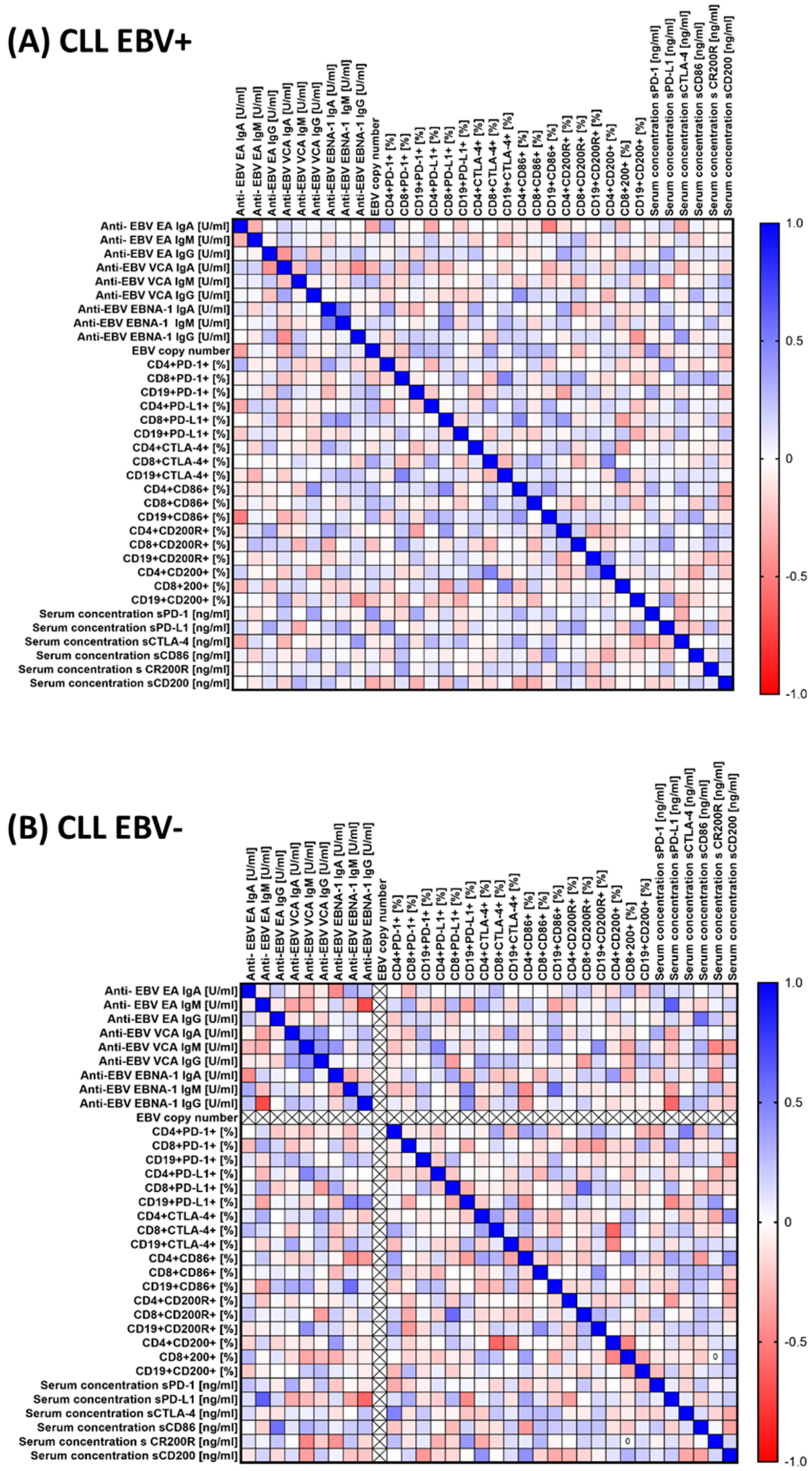

Figure S1. Spearman rank correlation analysis of patients with CLL EBV+ (A) and CLL EBV- (B).

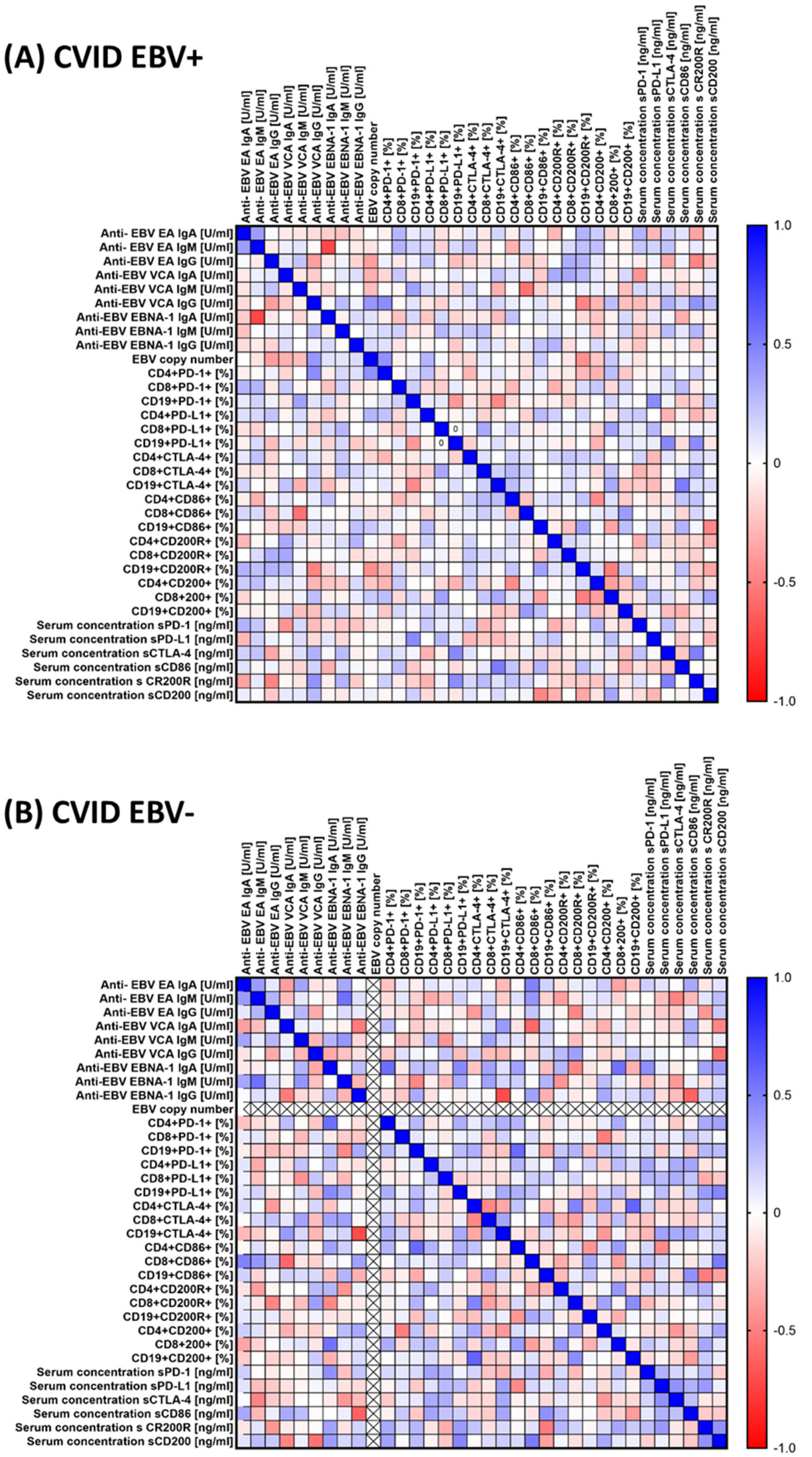

Figure S2. Spearman rank correlation analysis of patients with CVID EBV+ (A) and CVID EBV- (B).

Table S6. Sperman rank correlations for CLL EBV+ patients

Parameters	R Spearman	t(N-2)	p- value
EA IgA [U/ml] & CD19+CD86+ [%]	-0.50029	-3.3691	0.001889*
VCA IgA & EBNA IgG [U/ml]	-0.446847	-2.91249	0.006293*
EA IgG & VCA IgA[U/ml]	-0.421622	-2.71122	0.010434*
EBNA IgG [U/ml] & CD19+CD200+ [%]	-0.407979	-2.60562	0.01351*
CD8+PD-L1+ & CD8+200+ [%]	-0.371686	-2.33453	0.025611*
EA IgA [U/ml] & EBV copy number	-0.364221	-2.28039	0.028975*
CD19+PD-1+ & CD4+CD200R+ [%]	-0.357662	-2.23324	0.032222*
EA IgA [U/ml] & CD4+PD-L1+ [%]	-0.342386	-2.12487	0.040947*
CD4+CD86+ [%] & Serum concentration sCD200 [ng/ml]	-0.332068	-2.05275	0.047857*
CD4+CTLA-4+ & CD8+200+ [%]	-0.331532	-2.04903	0.04824*
EBNA IgM [U/ml] & Serum concentration sPD-L1 [ng/ml]	0.329215	2.03296	0.049923*
EBNA IgA [U/ml] & CD4+CD200R+ [%]	0.329472	2.03474	0.049735*
EBV copy number & CD8+CTLA-4+ [%]	0.335436	2.0762	0.045506*
EA IgG[U/ml] & CD4+CD200R+ [%]	0.336852	2.08608	0.044545*
EA IgG [U/ml] & Serum concentration sPD-L1 [ng/ml]	0.336937	2.08668	0.044488*
CD8+CTLA-4+ & CD8+CD86+ [%]	0.339383	2.10379	0.042869*
CD19+CD200R+ & CD4+CD200+ [%]	0.341206	2.11657	0.041694*
EBNA IgA & CD8+PD-L1+	0.341721	2.12019	0.041367*
CD8+PD-1+ [%] & Serum concentration s CD200R+ [%]	0.34275	2.12743	0.040719*
VCA IgA & VCA IgG [U/ml]	0.348005	2.1645	0.037539*
CD4+PD-1+ [%] & Serum concentration sPD-L1 [ng/ml]	0.348005	2.1645	0.037539*
CD8+CD200R+ [%]& Serum concentration sPD-L1 [ng/ml]	0.351609	2.19006	0.035477*
VCA IgG [U/ml] & Serum concentration sPD-1 [ng/ml]	0.351866	2.19188	0.035334*
EBNA IgA [U/ml] & CD4+PD-1+ [%]	0.363086	2.27221	0.029516*
CD19+CD200+ [%] & Serum concentration sPD-L1[ng/ml]	0.366538	2.29714	0.027894*
CD8+PD-L1+ & CD4+CD200R+ [%]	0.381774	2.40854	0.021585*
EBNA IgG [U/ml] & Serum concentration sCTLA-4 [ng/ml]	0.385378	2.43522	0.02028*
CD4+CD86+ & CD19+CD86+ [%]	0.393616	2.4967	0.017543*
EBV copy number & Serum concentration sPD-1 [ng/ml]	0.397426	2.52538	0.016386*
EBNA IgM [U/ml] & CD8+PD-L1+ [%]	0.409781	2.61944	0.013065*
VCA IgG [U/ml] & CD4+CD86+ [%]	0.411996	2.63648	0.012534*
CD19+CTLA-4+ & CD8+200+ [%]	0.446676	2.91109	0.006315*
CD8+PD-L1+ & CD4+CD86+ [%]	0.45344	2.96648	0.005479*
CD8+PD-1+ & CD19+CTLA-4+ [%]	0.475798	3.15427	0.003357*
CD8+CTLA-4+ & CD4+CD200+ [%]	0.482139	3.20894	0.002904*
EBNA IgA & EBNA IgM [U/ml]	0.507369	3.43315	0.001586*

* statistically significant results

Table S7. Spearman rank correlations for CLL EBV- patients

Parameters	R Spearman	t(N-2)	p-value
EA IgM & EBNA IgG [U/ml]	-0.71031	-4.73319	0.000101*
CD8+CTLA-4+ & CD4+CD200+ [%]	-0.60404	-3.55507	0.001773*
EBNA IgG [U/ml] & Serum concentration sPD-L1 [ng/ml]	-0.59391	-3.46252	0.002214*
VCA IgM [U/ml] & Serum concentration s CR200R [ng/ml]	-0.47924	-2.56106	0.017814*
CD4+CD200+ & CD8+200+ [%]	-0.47315	-2.51906	0.019536*
CD19+CTLA-4+ & CD4+CD200+ [%]	-0.46977	-2.49597	0.020547*
EA IgA & EBNA IgA [U/ml]	-0.46097	-2.43645	0.023384*
CD19+PD-L1+ [%] & Serum concentration sPD-L1 [ng/ml]	-0.46097	-2.43645	0.023384*
EBNA IgM [U/ml]& CD4+CD86+ [%]	-0.45271	-2.38139	0.026327*
EBNA IgA [U/ml] & Serum concentration sCD200R [ng/ml]	-0.43009	-2.23455	0.035926*
CD19+PD-1+ [%] & Serum concentration sCD200 [ng/ml]	-0.40522	-2.07897	0.049486*
VCA IgM & VCA IgG [U/ml]	0.404871	2.07684	0.0497*
VCA IgM & EBNA IgA[U/ml]	0.411048	2.11492	0.045998*
CD8+CD86+ & CD19+CD200R+ [%]	0.427049	2.21519	0.037406*
VCA IgM [U/ml] & CD19+CD200R+ [%]	0.427919	2.22071	0.036979*
CD4+CTLA-4+[%] & Serum concentration sCD200 [ng/ml]	0.429565	2.23118	0.03618*
CD4+CD86+ [%] & Serum concentration sCD200 [ng/ml]	0.431398	2.24288	0.035306*
EBNA IgG [U/ml]& CD19+PD-L1+ [%]	0.431833	2.24565	0.035101*
VCA IgM [U/ml]& CD4+PD-L1+ [%]	0.468363	2.48639	0.020981*
EBNA IgM [U/ml]& CD19+PD-L1+ [%]	0.472711	2.51608	0.019664*
CD4+PD-1+ [%]& Serum concentration sCTLA-4 [ng/ml]	0.493043	2.65812	0.014364*
EA IgG [U/ml]& Serum concentration sCD86 [ng/ml]	0.558261	3.15606	0.004582*
EBNA IgM [U/ml]& CD19+CD86+ [%]	0.563478	3.19919	0.00414*
CD8+PD-L1+ & CD8+CD200R+ [%]	0.579073	3.33151	0.003027*
EA IgM [U/ml]& Serum concentration sPD-L1 [ng/ml]	0.625054	3.75587	0.001092*

* statistically significant results

Table S8. Spearman rank correlations for CVID EBV+ patients

Parameters	R Spearman	t(N-2)	p-value
EA IgM & EBNA IgA [U/ml]	-0.71918	-5.47701	0.000008*
VCA IgM [U/ml]& CD8+CD86+ [%]	-0.55256	-3.50807	0.001544*
CD19+CD200R+ & CD8+200+ [%]	-0.50345	-3.08324	0.004567*
EA IgG [U/ml] & Serum concentration s CD200R [ng/ml]	-0.48459	-2.93141	0.006651*
VCA IgG [U/ml]& CD19+CD200R+ [%]	-0.48254	-2.91519	0.006921*
CD19+CD86+ [%] & Serum concentration sCD200 [ng/ml]	-0.47102	-2.82546	0.008609*
CD19+PD-1+ & CD19+CTLA-4+ [%]	-0.46123	-2.75065	0.010306*
CD4+CD86+ & CD4+CD200+ [%]	-0.45076	-2.67207	0.012424*

EBV copy number& CD19+CD200R+ [%]	-0.43804	-2.57844	0.015475*
VCA IgA[U/ml] & Serum concentration sPD-1 [ng/ml]	-0.42114	-2.45693	0.020471*
CD19+PD-1+ & CD19+PD-L1+ [%]	-0.40783	-2.36354	0.025277*
EA IgG & VCA IgG [U/ml]	-0.38421	-2.20203	0.036067*
CD4+CD200+ & CD8+200+ [%]	-0.38403	-2.20082	0.036161*
EA IgG [U/ml]& EBV copy number	-0.38198	-2.18709	0.037249*
EA IgA [U/ml] & Serum concentration sCR200R [ng/ml]	-0.36689	-2.08695	0.046116*
VCA IgM [U/ml] & CD19+PD-1+ [%]	0.363778	2.06652	0.04814*
CD8+PD-L1+ & CD8+200+ [%]	0.36845	2.0972	0.04513*
EA IgA & EA IgM [U/ml]	0.384025	2.20082	0.036161*
CD8+CD86+ & CD19+CD200+	0.394699	2.2731	0.030889*
VCA IgG [U/ml]& EBV copy number	0.407786	2.36322	0.025294*
VCA IgG [U/ml]& Serum concentration sCR200R [ng/ml]	0.423184	2.47149	0.019802*
EBV copy number & CD4+PD-1+ [%]	0.434038	2.54937	0.016555*
VCA IgG [U/ml] & CD4+PD-1+ [%]	0.446051	2.63716	0.013489*
CD19+PD-L1+ [%] & Serum concentration s CD200R [ng/ml]	0.447213	2.64575	0.013219*
CD19+PD-1+ [%] & Serum concentration sPD-L1 [ng/ml]	0.451207	2.67538	0.012327*
CD19+PD-L1+ [%] & Serum concentration sCTLA-4 [ng/ml]	0.459399	2.73681	0.010653*
Serum concentration sCTLA-4 & Serum concentration s CD200R [ng/ml]	0.491267	2.98452	0.005837*
CD19+CTLA-4+ [%]& Serum concentration sCD86 [ng/ml]	0.511069	3.14625	0.0039*

* statistically significant results

Table S9. Spearman rank correlations for CVID EBV- patients

Parameters	R Spearman	t(N-2)	p-value
EBNA IgG [U/ml] & CD19+CTLA-4+ [%]	-0.70412	-4.43462	0.000255
EBNA IgG [U/ml] & Serum concentration sCD86 [ng/ml]	-0.60887	-3.43254	0.002636
VCA IgA [U/ml] & CD8+CD86+ [%]	-0.59119	-3.2781	0.003761
VCA IgG [U/ml] & Serum concentration sCD200 [ng/ml]	-0.55054	-2.94926	0.00793
VCA IgA & EBNA IgG [U/ml]	-0.51553	-2.69061	0.014064
CD19+CD86+ [%] & Serum concentration s CD200R [ng/ml]	-0.51511	-2.68763	0.014156
CD8+PD-1+ & CD4+CD200+ [%]	-0.50536	-2.61912	0.016432
EA IgM [U/ml] & Serum concentration sCTLA-4 [ng/ml]	-0.48305	-2.46721	0.02277
VCA IgA [U/ml]& Serum concentration sCD200 [ng/ml]	-0.47826	-2.43544	0.024357
EA IgG [U/ml] & CD8+CD200R+ [%]	-0.476	-2.42056	0.025135
EBNA IgA [U/ml] & CD8+CD200R+ [%]	-0.46923	-2.37628	0.02759
EBNA IgM [U/ml] & CD19+PD-1+ [%]	-0.46923	-2.37628	0.02759
CD4+CTLA-4+ & CD8+CTLA-4+ [%[	-0.46358	-2.3398	0.029778
CD4+CD86+ [%] & Serum concentration sPD-L1 [ng/ml]	-0.46132	-2.32531	0.030691
Serum concentration sPD-L1 & Serum concentration sCTLA-4 [ng/ml]	0.424739	2.09815	0.048798
CD19+CD86+ [%]& Serum concentration sCD86 [ng/ml]	0.427563	2.11521	0.047159

Serum concentration s CR200R & Serum concentration sCD200 [ng/ml]	0.427563	2.11521	0.047159
EBNA IgA [U/ml]& Serum concentration sPD-1 [ng/ml]	0.431959	2.14192	0.044692
CD8+CD200R+ & CD19+CD200+ [%]	0.446766	2.23327	0.037117
EBNA IgA [U/ml] & CD19+PD-L1+ [%]	0.456804	2.2965	0.032583
CD19+PD-L1+ [%] & Serum concentration sCD200 [ng/ml]	0.472614	2.39835	0.02634
CD4+CTLA-4+ & CD8+CD200R+ [%]	0.480519	2.45038	0.023598
EA IgA [U/ml] & CD8+CD86+ [%]	0.487295	2.4956	0.021434
CD8+CD86+[%] & Serum concentration sCD200 [nf/ml]	0.516657	2.69865	0.013819
EBNA IgA [U/ml] & CD8+200+ [%]	0.549562	2.94178	0.008064
EA IgM & EBNA IgM [U/ml]	0.555775	2.98978	0.00724
EBNA IgA [U/ml] & CD4+PD-1+ [%]	0.564088	3.05515	0.006248
CD19+PD-1+ & CD4+CD86+ [%]	0.57764	3.16466	0.004874
CD4+CTLA-4+ & CD19+CD200+ [%]	0.607173	3.4174	0.00273

* statistically significant results

Table S10. ROC curve analysis

Parameter		CLL EBV+ vs. CLL EBV-	CLL EBV+ vs. CVID EBV+	CLL EBV+ vs. HV EBV-	CVID EBV+ vs. CVID EBV-	CVID EBV+ vs. HV EBV-
Anti-EBV EA IgA	AUC	1,000	0,809	1,000	1,000	1,000
	SE	0,000	0,054	0,000	0,000	0,000
	-95%	1,000	0,704	1,000	1,000	1,000
	+95%	1,000	0,915	1,000	1,000	1,000
	z statistic	-	5,75	-	-	-
	p value	0,000	0,000	0,000	0,000	0,000
Anti-EBV EA IgM	AUC	0,631	0,415	0,609	0,697	0,679
	SE	0,071	0,074	0,071	0,079	0,075
	-95%	0,492	0,271	0,470	0,543	0,531
	+95%	0,771	0,560	0,748	0,851	0,826
	z statistic	1,848	-1,147	1,538	2,502	2,377
	p value	0,0646	0,2513	0,124	0,012	0,0174
Anti-EBV EA IgG	AUC	1,000	0,849	1,000	1,000	1,000
	SE	0,000	0,046	0,000	0,000	0,000
	-95%	1,000	0,760	1,000	1,000	1,000
	+95%	1,000	0,938	1,000	1,000	1,000
	z statistic	-	7,655	-	-	-
	p value	0,000	0,000	0,000	0,000	0,000
Anti-EBV VCA IgA	AUC	1,000	0,835	1,000	1,000	1,000
	SE	0,000	0,049	0,000	0,000	0,000
	-95%	1,000	0,740	1,000	1,000	1,000
	+95%	1,000	0,931	1,000	1,000	1,000
	z statistic	-	6,871	-	-	-
	p value	0,000	0,000	0,000	0,000	0,000
Anti-EBV VCA IgM	AUC	1,000	0,590	1,000	1,000	1,000
	SE	0,000	0,073	0,000	0,000	0,000
	-95%	1,000	0,446	1,000	1,000	1,000
	+95%	1,000	0,733	1,000	1,000	1,000
	z statistic	-	1,228	-	-	-
	p value	0,000	0,219	0,000	0,000	0,000
Anti-EBV VCA IgG	AUC	0,979	0,886	1,000	0,645	0,971
	SE	0,015	0,04	0,000	0,077	0,017
	-95%	0,951	0,808	1,000	0,494	0,937
	+95%	1,00	0,964	1,000	0,797	1,00
	z statistic	32,871	9,67	-	1,883	27,326
	p value	0,000	0,000	0,000	0,0597	0,000

Anti-EBV EBNA-1 IgA	AUC	1,000	0,877	1,000	1,000	1,000
	SE	0,000	0,041	0,000	0,000	0,000
	-95%	1,000	0,797	1,000	1,000	1,000
	+95%	1,000	0,957	1,000	1,000	1,000
	z statistic	-	9,230	-	-	-
	p value	0,000	0,000	0,000	0,000	0,000
Anti-EBV EBNA-1 IgM	AUC	0,754	0,577	0,894	0,867	0,739
	SE	0,061	0,075	0,037	0,05	0,067
	-95%	0,634	0,429	0,820	0,769	0,608
	+95%	0,874	0,724	0,967	0,966	0,870
	z statistic	4,148	1,020	10,560	7,336	3,583
	p value	0,000	0,307	0,000	0,000	0,003
Anti-EBV EBNA-1 IgG	AUC	1,000	0,594	1,000	1,000	1,000
	SE	0,000	0,072	0,000	0,000	0,000
	-95%	1,000	0,452	1,000	1,000	1,000
	+95%	1,000	0,735	1,000	1,000	1,000
	z statistic	-	1,299	-	-	-
	p value	0,000	0,193	0,000	0,000	0,000
CD4+PD-1+[%]	AUC	1,000	0,908	1,000	0,965	1,000
	SE	0,000	0,037	0,000	0,021	0,000
	-95%	1,000	0,835	1,000	0,924	1,000
	+95%	1,000	0,982	1,000	1,000	1,000
	z statistic	-	10,900	-	22,398	-
	p value	0,000	0,000	0,000	0,000	0,000
CD8+PD-1+[%]	AUC	1,000	0,250	1,000	1,000	1,000
	SE	0,000	0,06	0,000	0,000	0,000
	-95%	1,000	0,132	1,000	1,000	1,000
	+95%	1,000	0,369	1,000	1,000	1,000
	z statistic	-	-4,125	-	-	-
	p value	0,000	0,000	0,000	0,000	0,000
CD19+PD-1+[%]	AUC	0,986	0,978	1,000	1,000	1,000
	SE	0,011	0,014	0,000	0,000	0,000
	-95%	0,964	0,951	1,000	1,000	1,000
	+95%	1,000	1,000	1,000	1,000	1,000
	z statistic	43,047	34,353	-	-	-
	p value	0,000	0,000	0,000	0,000	0,000
CD4+PD-L1+[%]	AUC	1,000	0,942	1,000	1,000	1,000
	SE	0,000	0,025	0,000	0,000	0,000
	-95%	1,000	0,893	1,000	1,000	1,000
	+95%	1,000	0,992	1,000	1,000	1,000
	z statistic	-	17,524	-	-	-

	p value	0,000	0,000	0,000	0,000	0,000
CD8+PD-L1+[%]	AUC	1,000	0,770	1,000	1,000	1,000
	SE	0,000	0,058	0,000	0,000	0,000
	-95%	1,000	0,657	1,000	1,000	1,000
	+95%	1,000	0,884	1,000	1,000	1,000
	z statistic	-	4,669	-	-	-
	p value	0,000	0,000	0,000	0,000	0,000
CD19+PD-L1+[%]	AUC	1,000	0,816	1,000	1,000	1,000
	SE	0,000	0,051	0,000	0,000	0,000
	-95%	1,000	0,716	1,000	1,000	1,000
	+95%	1,000	0,916	1,000	1,000	1,000
	z statistic	-	6,209	-	-	-
	p value	0,000	0,000	0,000	0,000	0,000
CD4+CTLA-4+[%]	AUC	1,000	0,839	1,000	1,000	1,000
	SE	0,000	0,047	0,000	0,000	0,000
	-95%	1,000	0,746	1,000	1,000	1,000
	+95%	1,000	0,931	1,000	1,000	1,000
	z statistic	-	7,177	-	-	-
	p value	0,000	0,000	0,000	0,000	0,000
CD8+CTLA-4+[%]	AUC	1,000	0,255	1,000	1,000	1,000
	SE	0,000	0,067	0,000	0,000	0,000
	-95%	1,000	0,123	1,000	1,000	1,000
	+95%	1,000	0,386	1,000	1,000	1,000
	z statistic	-	-3,657	-	-	-
	p value	0,000	0,0003	0,000	0,000	0,000
CD19+CTLA-4+[%]	AUC	1,000	0,000	1,000	1,000	1,000
	SE	0,000	0,000	0,000	0,000	0,000
	-95%	1,000	0,000	1,000	1,000	1,000
	+95%	1,000	0,0000	1,000	1,000	1,000
	z statistic	-	-	-	-	-
	p value	0,000	0,000	0,000	0,000	0,000
CD4+CD86+[%]	AUC	1,000	0,826	1,000	1,000	1,000
	SE	0,000	0,049	0,000	0,000	0,000
	-95%	1,000	0,730	1,000	1,000	1,000
	+95%	1,000	0,923	1,000	1,000	1,000
	z statistic	-	6,623	-	-	-
	p value	0,000	0,000	0,000	0,000	0,000
CD8+CD86+[%]	AUC	0,939	0,833	1,000	1,000	1,000
	SE	0,028	0,053	0,000	0,000	0,000
	-95%	0,884	0,730	1,000	1,000	1,000
	+95%	0,995	0,936	1,000	1,000	1,000

	z statistic	15,46	6,334	-	-	-
	p value	0,000	0,000	0,000	0,000	0,000
CD19+CD86+[%]	AUC	1,000	1,000	1,000	1,000	1,000
	SE	0,000	0,000	0,000	0,000	0,000
	-95%	1,000	1,000	1,000	1,000	1,000
	+95%	1,000	1,000	1,000	1,000	1,000
	z statistic	-	-	-	-	-
	p value	0,000	0,000	0,000	0,000	0,000
CD4+CD200R+[%]	AUC	1,000	0,991	1,000	1,000	0,934
	SE	0,000	0,0007	0,000	0,000	0,029
	-95%	1,000	0,977	1,000	1,000	0,877
	+95%	1,000	1,000	1,000	1,000	0,992
	z statistic	-	68,498	-	-	14,894
	p value	0,000	0,000	0,000	0,000	0,000
CD8+CD200R+[%]	AUC	1,000	0,814	1,000	1,000	1,000
	SE	0,000	0,052	0,000	0,000	0,000
	-95%	1,000	0,711	1,000	1,000	1,000
	+95%	1,000	0,916	1,000	1,000	1,000
	z statistic	-	6,007	-	-	-
	p value	0,000	0,000	0,000	0,000	0,000
CD19+CD200R+[%]	AUC	1,000	0,809	0,519	1,000	0,238
	SE	0,000	0,053	0,073	0,000	0,063
	-95%	1,000	0,705	0,375	1,000	0,115
	+95%	1,000	0,914	0,662	1,000	0,362
	z statistic	-	5,801	0,252	-	-4,144
	p value	0,000	0,000	0,800	0,000	0,000
CD4+CD200+[%]	AUC	1,000	0,984	1,000	1,000	1,000
	SE	0,000	0,011	0,000	0,000	0,000
	-95%	1,000	0,963	1,000	1,000	1,000
	+95%	1,000	1,000	1,000	1,000	1,000
	z statistic	-	44,950	-	-	-
	p value	0,000	0,000	0,000	0,000	0,000
CD8+CD200+[%]	AUC	1,000	0,587	1,000	1,000	1,000
	SE	0,000	0,077	0,000	0,000	0,000
	-95%	1,000	0,436	1,000	1,000	1,000
	+95%	1,000	0,737	1,000	1,000	1,000
	z statistic	-	1,125	-	-	-
	p value	0,000	0,260	0,000	0,000	0,000
CD19+CD200+[%]	AUC	1,000	0,907	1,000	0,916	0,853
	SE	0,000	0,04	0,000	0,037	0,048
	-95%	1,000	0,828	1,000	0,843	0,760

	+95%	1,000	0,986	1,000	0,989	0,946
	z statistic	-	10,107	-	11,176	7,438
	p value	0,000	0,000	0,000	0,000	0,000
Serum concentration sPD-1	AUC	0,917	1,000	1,000	1,000	1,000
	SE	0,034	0,000	0,000	0,000	0,000
	-95%	0,850	1,000	1,000	1,000	1,000
	+95%	0,983	1,000	1,000	1,000	1,000
	z statistic	12,334	-	-	-	-
	p value	0,000	0,000	0,000	0,000	0,000
Serum concentration sPD-L1	AUC	0,972	0,972	1,000	1,000	1,000
	SE	0,027	0,027	0,000	0,000	0,000
	-95%	0,919	0,919	1,000	1,000	1,000
	+95%	1,000	1,000	1,000	1,000	1,000
	z statistic	17,241	17,241	-	-	-
	p value	0,000	0,000	0,000	0,000	0,000
Serum concentration sCTLA-4	AUC	0,898	1,000	1,000	1,000	1,000
	SE	0,051	0,000	0,000	0,000	0,000
	-95%	0,797	1,000	1,000	1,000	1,000
	+95%	0,999	1,000	1,000	1,000	1,000
	z statistic	7,735	-	-	-	-
	p value	0,000	0,000	0,000	0,000	0,000
Serum concentration sCD86	AUC	1,000	1,000	1,000	0,700	1,000
	SE	0,000	0,000	0,000	0,078	0,000
	-95%	1,000	1,000	1,000	0,546	1,000
	+95%	1,000	1,000	1,000	0,854	1,000
	z statistic	-	-	-	2,55	-
	p value	0,000	0,000	0,000	0,011	0,000
Serum concentration sCD200R	AUC	1,000	1,000	1,000	1,000	1,000
	SE	0,000	0,000	0,000	0,000	0,000
	-95%	1,000	1,000	1,000	1,000	1,000
	+95%	1,000	1,000	1,000	1,000	1,000
	z statistic	-	-	-	-	-
	p value	0,000	0,000	0,000	0,000	0,000
Serum concentration sCD200	AUC	1,000	0,811	1,000	1,000	1,000
	SE	0,000	0,051	0,000	0,000	0,000
	-95%	1,000	0,711	1,000	1,000	1,000
	+95%	1,000	0,912	1,000	1,000	1,000
	z statistic	-	6,073	-	-	-
	p value	0,000	0,000	0,000	0,000	0,000
